# Metacell-based differential expression analysis identifies cell type specific temporal gene response programs in COVID-19 patient PBMCs

**DOI:** 10.1038/s41540-024-00364-2

**Published:** 2024-04-05

**Authors:** Kevin O’Leary, Deyou Zheng

**Affiliations:** 1 Department of Genetics, Albert Einstein College of Medicine, Bronx, NY USA; 2 Department of Neurology, Albert Einstein College of Medicine, Bronx, NY USA; 3 Department of Neuroscience, Albert Einstein College of Medicine, Bronx, NY USA

**Keywords:** Software, Time series

## Abstract

By profiling gene expression in individual cells, single-cell RNA-sequencing (scRNA-seq) can resolve cellular heterogeneity and cell-type gene expression dynamics. Its application to time-series samples can identify temporal gene programs active in different cell types, for example, immune cells’ responses to viral infection. However, current scRNA-seq analysis has limitations. One is the low number of genes detected per cell. The second is insufficient replicates (often 1-2) due to high experimental cost. The third lies in the data analysis—treating individual cells as independent measurements leads to inflated statistics. To address these, we explore a new computational framework, specifically whether “metacells” constructed to maintain cellular heterogeneity within individual cell types (or clusters) can be used as “replicates” for increasing statistical rigor. Toward this, we applied SEACells to a time-series scRNA-seq dataset from peripheral blood mononuclear cells (PBMCs) after SARS-CoV-2 infection to construct metacells, and used them in maSigPro for quadratic regression to find significantly differentially expressed genes (DEGs) over time, followed by clustering expression velocity trends. We showed that such metacells retained greater expression variances and produced more biologically meaningful DEGs compared to either metacells generated randomly or from simple pseudobulk methods. More specifically, this approach correctly identified the known ISG15 interferon response program in almost all PBMC cell types and many DEGs enriched in the previously defined SARS-CoV-2 infection response pathway. It also uncovered additional and more cell type-specific temporal gene expression programs. Overall, our results demonstrate that the metacell-pseudoreplicate strategy could potentially overcome the limitation of 1-2 replicates.

## Introduction

Single-cell RNA sequencing (scRNA-seq) is a powerful tool that can detect distinct gene expression dynamics in different cell types within a sample^1,2^. One can apply the analysis to time-series samples for the identification of temporal changes in gene expression and pathway activities within each cell type. To do this, a currently common practice is to use each cell as a statistically independent “observation” for determining gene expression change between time points. Statistically, this is not rigorous because cells in the same biological sample do not really represent independent measurements, but have intrinsic correlations^3^. Pseudobulking has been proposed to overcome this, where gene read counts for all cells of a cell type (or cluster) in a biological sample are aggregated. This approach also has an advantage in increasing gene coverage, as a relatively low number of genes are detected per cell by current scRNA-seq analysis approaches^4^. The strategy, however, brings up the problem of low numbers of replicates in scRNA-seq studies due to the high cost of library preparation and sequencing^5^. In addition, simply aggregating reads in all cells of a type may erase the bona fide heterogeneity (or variation) in a cell type (or cluster). In this study, we propose the use of “metacells” to circumnavigate these problems. A metacell represents the transcriptomes of a group of highly similar cells^6^. Multiple methods and algorithms exist to create them^6–8^; however, the single-cell aggregation of cell states (SEACells) algorithm has an advantage in retaining heterogeneity within each cell cluster^9^, resulting in metacells representing different transcriptomic states. It achieves this by constructing a k-nearest neighbor (KNN) graph representing the cell-to-cell similarity in the scRNA-seq data, analyzing the cell density on the graph, and then using archetypal analysis to partition cells to metacells that can represent cells at diverse regions of the KNN graph^9^. We thus decided to investigate if the metacells from SEACells can be used as pseudo-replicates (referred as “metareplicates”) in statistical methods that were developed for time-series data from bulk tissues (vs single cells). Considering the continued importance of understanding the diverse ways in which the immune system responds to severe acute respiratory syndrome coronavirus 2 (SARS-CoV-2), we further decided to test the approach with a time series dataset derived from coronavirus disease 2019 (COVID-19) patients following symptom onset^10^.

SARS‑CoV‑2, the strain of coronavirus responsible for the COVID-19 pandemic^11,12^, continues to infect hundreds of thousands of people around the globe. To date, over 7 million confirmed deaths have been recorded as a consequence of SARS-CoV-2 infection^13^. The desire to understand the mechanisms behind SARS-CoV-2 infection and host defense, especially as it relates to its transmissibility^14,15^ and severity^16^, has prompted a vast amount of research in the field of immunology and beyond^17,18^. One of many topics of interest concerns gene programs within cell types that respond to SARS-CoV-2, specifically peripheral blood mononuclear cells (PBMCs), which are round nucleus containing blood cells such as dendritic cells, lymphocytes, natural killer cells (NKs), or monocytes^19^. Because PBMCs are responsible for responding to and eliminating viral infections such as SARS-CoV-2, it is important to understand the transcriptomic basis of this process. Researchers have compared gene expression in PBMC cell types between COVID-19 patients and controls using bulk RNA sequencing^20^. Others have implemented scRNA-seq^21,22^, which provides greater resolution at the cellular level, especially as it relates to deducing cell type-specific responses to SARS-CoV-2 infection. Some have even performed time series scRNA-seq analysis of COVID-19 progression. While these studies have provided valuable information related to cell type-specific changes in expression through time, they were limited by the issues of small replicates as discussed above. For example, some time points in the PBMC scRNA-seq data that we planned to analyze have only one replicate. Consequently, the authors had to bin samples of time points to increase statistical power^10^; so did in other studies^20,23,24^. In addition to this computational difference, the scope and focus of our current study are also different from those in the original report^10^, e.g., the original authors focused on the response difference between COVID-19 infection and flu and did not study the velocity of the expression changes. The authors of SEACells also studied SARS-CoV-2 gene responses in PBMCs with a different dataset^21^, but focused on CD4 T cells and only analyzed a few metacells that could be assigned to specific time points^9^. This differs from our study in that we analyzed metacells representing 10 discrete points in time and in many PBMC cell types.

In short, using the SEACells alogorithm, we created metacells that retained hetergeneity and likely reprensented cell states within each cell type and used them as metareplicates. This resulted in up to 12 replicates for some time points and thus provided the statistical power necessary to resolve signficiant changes in expression through time. With that, we performed a strict statistical analysis through a greater number of time points than any other COVID-19 time-series scRNA-seq study to date. To accomplish this, we subset all cells based on time since symptom onset and then used the SEACells algorithm to create metacells. maSigPro^25^ was used for quadratic regression to find significantly differentially expressed genes (DEGs) through time, due to its robust statistical base, its flexibility with defining degrees of regression, and widespread use for time series analysis. Additionally, quadratic regression was used because we did not want to capture cyclical variation, rather we hoped to find broader changes in expression through four weeks of COVID-19 symptoms. We further classified all DEGs by expression velocity trend based on fitted expression curves and their dynamic derivates. With this approach, we identified *ISG15* as a DEG through time when PBMC cell types were analyzed together. When cell types were analyzed independently, however, we found many immune system-related DEGs, which enabled us to expand upon previous reports of certain gene programs and their relevance to SARS-CoV-2 immune response.

## Results

### Finding DEGs through time with either pseudobulking or individual cell-based methods

To characterize the dynamics of cell type gene programs in the PBMCs in response to SARS-CoV-2 infection, we first applied a pseudobulking approach by aggregating scRNA-seq reads for individual genes, for either all PBMC cells together or each of the cell types separately, for each sample. The samples and scRNA-seq data were collected at 10-time points representing postsymptom onset days, from day 3 to day 28 by Zhu et al., as described previously^10^. This generated timeseries pseudobulk RNA-seq data with 1 to 3 replicates, which were then used to identify genes exhibiting significant expression changes along the postinfection period by maSigPro. The regression ANOVA analysis did not find any DEGs when PBMCs were not separated into cell types but found a few DEGs for some cell types (1 for CD8/CD4 T cells, 1 for NKs, 2 for naïve B cells, 2 for XCL+ NKs, and 3 for plasma cells) (Supplementary Figure 1). However, most of the DEGs exhibited the same expression trend, suggesting model overfitting due to outliers and low replicates. We also performed the maSigPro analysis at the level of individual cells. Although many genes with FDR (derived from ANOVA p-values) < 0.05 existed, their R^2^ values (coefficient for regression fitting, see Methods) were all far below the 0.5 cutoff, an indication of inflated p-values (hundreds of cells) and poor model fitting (most points far away from the regression fitting curves). We thus concluded that both methods were not appropriate for the analysis.

### Characterizing DEGs through time using metacells as replicates

**Fig. 1:**
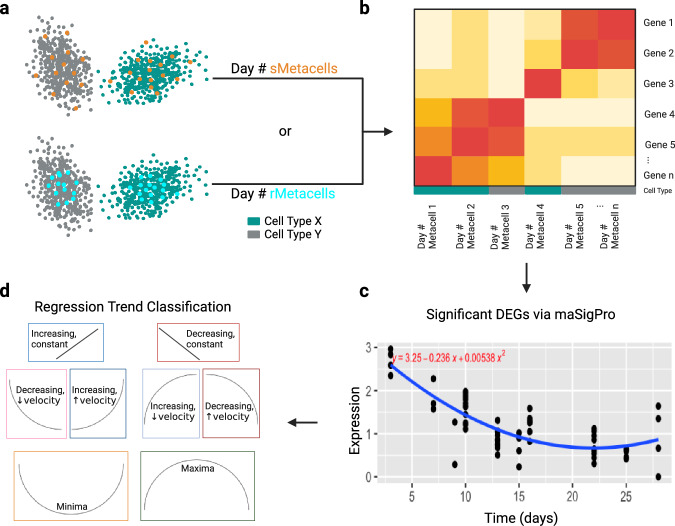
Summary of metacell generation and usage. **a** An example of metacells generated using the SEACells algorithm (sMetacells) and random single cells (rMetacells) for a time point. An example of the distribution of sMetacells (orange dots) and rMetacells (blue) are shown with overlaying all cells of a particular type (either grey or blue). **b** The gene expression of each metacell was computed from the average of the normalized expression amongst all single cells assigned to it. **c** After the generation of metacells for each time point, quadratic regression was performed for each gene. An example of a significantly changing gene is shown here. **d** One of eight expression velocity trends was assigned to each DEG. For the example in C, the trend would be “Decreasing, ↓ velocity” for decreasing expression with decreasing expression velocity.

We reasoned that using metacells to construct computational replicates (referred as “metareplicates”) may allow us to mitigate false positives and overfitting in the pseudobulk approach. To test this, we generated metacells from the scRNA-seq data for samples in each of the 10 time points independently using two different methods: SEACells and random selection (Fig. 1; see Methods). The resultant metacells were referred as “sMetacells” and “rMetacells”, respectively. Given that the SEACells algorithm retains heterogeneity to capture cell states within specific cell types, we expected that its metacells would introduce variation within individual time points, lead to fewer DEGs through time and be less prone to overfitting. We therefore compared the numbers of DEGs determined for these two methods (Table 1). We excluded all cell types with fewer than 500 total cells to avoid more extreme cases of overfitting for both methods since fewer cells would lead to fewer metacells (and thus too few metareplicates). After that, the average number of metareplicates per time point for each cell type using the SEACells algorithm was 3.18. For rMetacells, three metareplicates were created. The average number of cells assigned to each metacell was 71.6 for sMetacells and predetermined to be 20 for rMetacells (see Methods).

**Table 1:** Comparison of metareplicates from sMetacells and rMetacells and DEGs computed from them

	Metacell Method	sMetacells	rMetacells
A, Summary of Metacells	Metareplicates Per Time Point	3.2	3
	Avg # of Cells Assigned to Metacell	71.6	20
	Avg Variance Across Gene	0.041	0.0097
	Avg # Genes in Metacells	8440 (*n* = 370)	5930 (*n* = 450)
B, DEG numbers	All PBMCs without separating to cell types	1	10
	Cytotoxic CD8 T cells	41	31
	Naïve T cells	22	22
	NKs	32	43
	Activated CD4 T cells	128	64
	Naïve B	25	91
	Plasma	7	120
	Memory B	9	57
	XCL+ NKs	13	79
	MAIT	61	49
	Cycling T cells	332	633
	Total unique DEGs	580	1034

A, Replicates per time point, # cells per metacell, average variance, and average # of genes detected. B, The number of DEGs through time using quadratic regression at FDR < 0.05 and R^2^ > 0.5.

For each metacell type, we determined the standard deviation (SD) of each gene’s expression for each time point and used these values to calculate the mean SD (mSD) for all genes. Thus, we obtained mSD values for each time point and cell type for either rMetacells or sMetacells. sMetacells showed greater mSD, and therefore greater variance, for 71 out of 100 individual time points across cell types. Additionally, if these mSDs were further averaged among all time points and cell types, sMetacells still showed greater mSD (0.065) than rMetacells (0.041), a difference that was statistically significant (*p* = 2.76e–10, two-sided t-test). These results are summarized in Supplementary Table 1. Overall, this indicates that sMetacells provide bigger variances among metareplicates than rMetacells.

A more Important question is how the variances provided by metacells match the true or expected biological variances. Since at individual time points there were insufficient biological replicates (i.e., patients) to provide good estimate of sample variances, we decided to combine cells from all time points and computed gene SDs for each of the cell types, with pseudobulking, rMetacell, or sMetacell methods. The result indicated that the gene variances from sMetacells were very close to those from pseudobulking and significantly larger than those from rMetacells (Supplementary Figure 2), further supporting that sMetacells could be used as replicates. Interestingly, the average number of genes per metacell was also higher for sMetacells (8440) than rMetacells (5930) (*p* = 2.2e–16, two-sided t-test) (Table 1).

To address the potential effect that cell purity may have on sMetacell variance, we generated “pure” sMetacells, where >95% of single cells assigned to it were of the same type. We found that these pure sMetacells showed slightly lower gene SDs for most cell types compared to all sMetacells (“mixed purity”), as expected from less cell heterogeneity, but still higher than the rMetacells (Supplementary Figure 2). Note that among the 318 metacells used for this comparison, 184 were pure sMetacells. Considering this small difference, we proceeded to use all the sMetacells without further filtering by purity scores.

**Fig. 2:**
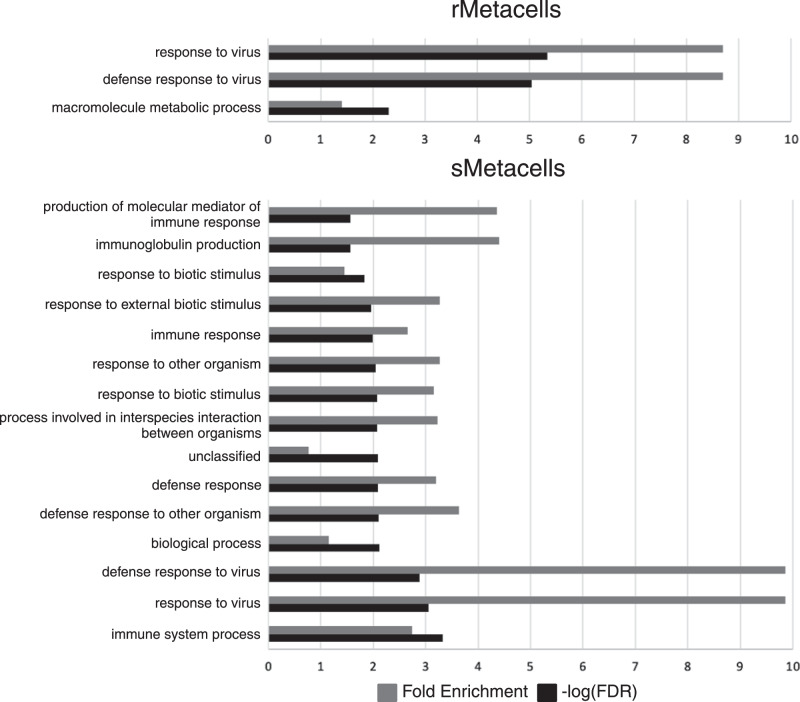
sMetacell-derived DEGs show stronger enrichment of biologically relevant pathways than those derived from random metacells. PANTHER GO-slim biological processes annotation data set was used to find enriched terms amongst DEGs through time from rMetacells and sMetacells.

Performing quadratic regression yielded more DEGs using rMetacells than sMetacells in most cell types, likely due to a higher degree of overfitting because of less variation across metareplicates (Table 1B). However, the difference between the total number of DEGs found using sMetacells vs rMetacells was not statistically significant (paired t-test). Of all the DEGs from the two methods, 140 were the same, leaving 440 and 894 unique to the sMetacell and rMetacell methods, respectively. To better understand the difference, we performed gene ontology (GO) enrichment analysis using all the DEGs identified from at least one of the cell types (FDR < 0.05 and R^2^ > 0.5) (Fig. 2). The results showed that the DEGs from the sMetacell method, despite fewer in number, were enriched with more significant GO terms, particularly those related to immune response. Additionally, for “defense response to virus” and “response to virus” terms, which were significant using DEGs from both methods, the fold enrichment scores were greater from data produced by sMetacells. Taken together, these results indicate that DEGs from sMetacells are more biologically relevant and less likely from statistical noise (i.e., false positives), e.g. overfitting due to underestimated variance by rMetacells. We therefore consider the metacells from the SEACells algorithm to be more appropriate metareplicates and continue to discuss the analyses and biological and disease implications from this method further in more details.

**Fig. 3:**
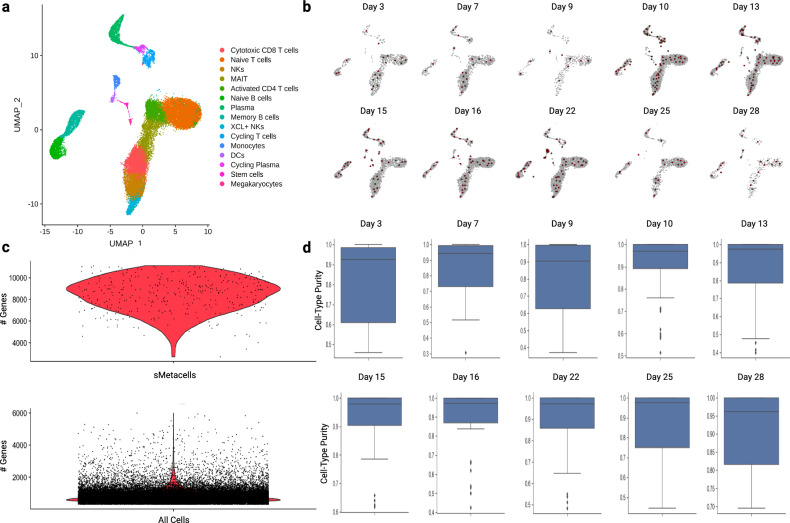
Summary of sMetacell output. **a** UMAP of 25,775 cells colored by cell type. **b** Metacell distribution across cell type space for each time point. Metacells are red while single cells are grey. **c** Violin plot of the number of genes detected for SEACell-generated metacells (top) compared to all single cells (bottom). **d** Box plots showing metacell purity for each day. The lower and upper hinges of the boxplots represent the first and third quartiles, the center line is the median, and the whiskers extend no further than 1.5 * interquartile range.

Supplementary Table 2 summarizes the number of samples, cells, and metacells for each time point using the SEACells algorithm. The cell identity of each metacell was assigned to the most frequent cell type among the individual single cells contributing to the metacell, using the metadata provided by Zhu et al. Figure 3a shows a UMAP representing the 25,775 cells from the COVID-19 patients and their assignment to one of the fifteen cell types. The SEACells algorithm performed exceptionally well in creating metacells that encompass the entirety of the cell type and state space for each time point (Fig. 3b). As expected, sMetacells had much higher numbers of genes detected (8840 on average) compared to single cells (814 on average) (Fig. 3c). The proportion of cells in each sMetacell that were from the same cell type were very high, indicating high sMetacell purity, with the average purity scores reaching 90% or higher (Fig. 3d).

**Fig. 4:**
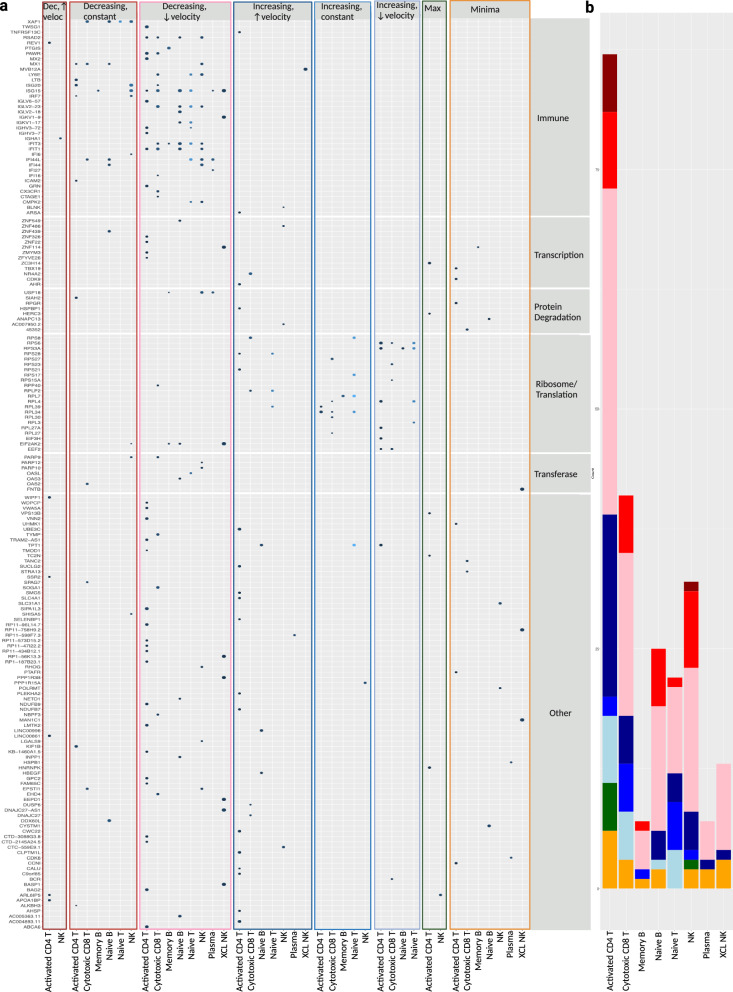
Summary of significant DEGs and expression trends by cell type. **a** Dot plot of all significant DEGs through time by trend type, protein class, and sMetacell type. A lighter blue dot corresponds to a lower p-value while a larger dot represents a larger R^2^. The trend “Decreasing, ↑ velocity” is abbreviated as “Dec, ↑ veloc”. **b** Summary of expression trends by metacell type. The y-axis corresponds to the frequency of significant DEGs through time for each cell type that correspond to a given trend pattern. **a**, **b** Red shades represent overall decreasing expression through time, blue shades are increasing, green is maxima (increasing then decreasing) and orange is minima (decreasing then increasing). The colors for the trends in **a** match the colors in **b**.

After eliminating cell types with low cell number or too few sMetacells (monocytes, DCs, cycling plasma, stem cells, and megakaryocytes) to be used as metareplicates in maSigPro analysis, we identified 328 unique DEGs through time with an R^2^ > 0.5 and FDR < 0.05, with some DEGs found in more than one cell type (Supplementary Table 3). We grouped the DEGs based on their functions and the cell type in which they were identified. Within each cell type, genes were further grouped according to their expression trends through time (Figs. 1, 4). We decided not to discuss DEGs for which the expression in one metacell was greater than zero while all others were zero, thus leading to overfitting of the model and less clear biological relevance. Likewise, visual inspection of clustered DEG trends for MAITs and cycling T cells (Supplementary Figure 3) found that a large group of DEGs were influenced by outliers, which led to overfitting. We therefore also eliminated these cell types from further discussion. With more cells, both of these post-filtering steps for DEGs are likely unnecessary. In the end, we kept 169 DEGs, with the greatest proportion coming from activated CD4 T cells, followed by cytotoxic CD8 T cells, NK cells, naïve B cells, Naïve T cells, XCL+ NKs, Plasma, and Memory B cells (Fig. 4b**)**.

At the level of general functional categories, the largest proportion of the DEGs were related to the immune system response (37), followed by genes related to ribosome/translation (22), transcription (13), protein degradation (8), and transferases (7). Since no other category contained more than 3-4 genes, genes unique to all those groups were classified as other; however, this does not imply that they have no role in response to SARS-CoV-2 infection. The functional classification results are summarized as a dot plot in Fig. 4a, along with the expression velocity patterns over times. 35/37 immune system-related genes showed one of three decreasing trends while just two increased through time (*TNFRSF13C* and *MVB12A* in activated CD4 T cells and XCL+ NKs, respectively). Genes implicated in transcriptional control and protein degradation showed a variety of increasing, decreasing, minima, and maxima trends. 20/22 genes related to the ribosome and/or translation increased through time while just two (*EIF2AK2*, and *RPP40*) decreased in multiple cell types. All transferase-related genes except *FNTB* in XCL+ NKs (minima trend) exhibited one of three decreasing trends. Among the remaining genes classified as other, there were a variety of increasing, decreasing, minima, and maxima trends; however, most decreased through time. The predominance of decreasing expression trends in genes involved in the immune response is consistent with Zhu et al.’s previous report, where they showed one group of genes with decreasing expression through different stages of COVID-19 were related to interferon signaling^10^. Additionally, they also showed an increase in expression of genes enriched with terms related to translation^10^, consistent with our results.

### Connecting many DEGs to genes previously implicated to SARS-CoV-2 response

**Fig. 5:**
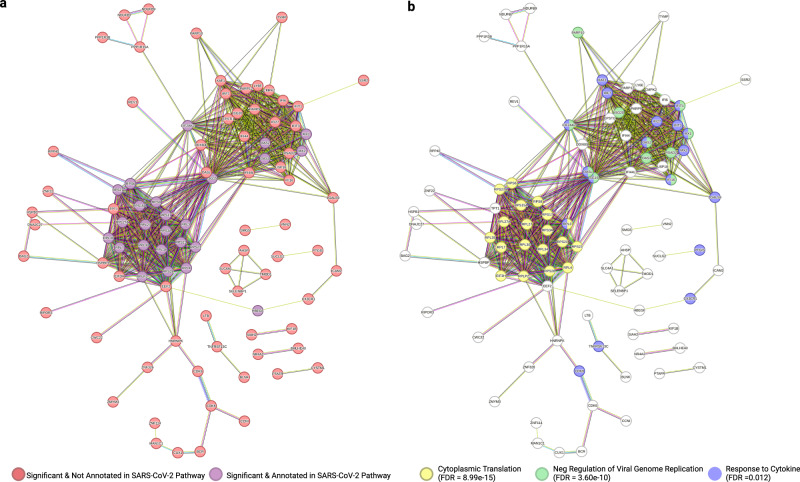
STRING protein interaction results. **a** STRING network colored by annotated vs unannotated KEGG COVID-19 pathway-related protein products. Red represents protein products from genes that are not annotated in the KEGG COVID-19 pathway, while purple represents those in this pathway. **b** STRING network colored by Biological Process GO Terms. GO terms were selected based on their ability to encompass 3 main clusters. Yellow represents Cytoplasmic Translation, blue represents Response to Cytokine, and green represents Negative Regulation of Viral Genome Replication.

Next, we asked how the 169 DEGs from our analysis are related to genes previously implicated in the COVID-19 pathway, based on KEGG. We input the protein names corresponding to these 169 genes into the StringDB to determine functional associations and colored the nodes by whether they are in the COVID-19 KEGG pathway (Fig. 5a). 93 of the 169 genes were determined to have a protein product that interacted with at least another protein from the input. Among these 93, 25 were previously annotated as part of the KEGG COVID-19 pathway. We also colored nodes by the protein’s affiliation with significant biological processes that capture the two largest clusters of connected proteins (Fig. 5b). Actual cluster identification prior to overlay with biological process identifiers can be found in Supplementary Figure 4. The analysis showed that Cytoplasmic Translation (FDR = 8.99e-15), Negative Regulation of Viral Genome Replication (FDR = 3.60e-10), and Response to Cytokine (FDR = 0.012) were among the significant GO terms corresponding to DEGs from these clusters. The proteins comprising the Cytoplasmic Translation cluster are EIF3H, RPLP2, RPL7, RPS28, RPL4, RPS3A, RPL3, RPS15A, RPS17, RPL30, RPL27, RPS21, RPL34, RPS23, RPS8, RPL39, RPL27A, RPS27, and RPS6. Among these, EIF3H was not previously annotated in the COVID-19 KEGG pathway. Proteins comprising the Negative Regulation of Viral Genome Replication are OASL, ISG15, EIF2AK2, OAS3, OAS2, MX1, RSAD2, ISG20, IFI16, and IFIT1. OASL, RSAD2, ISG20, IFI16, and IFIT1 were not previously annotated in the COVID-19 KEGG pathway. For the Response to Cytokine Pathway group, EIF2AK2, ISG15, OASL, MX1, OAS2, IFI16, and IFIT1 overlapped with the Negative Regulation of Viral Genome Replication group while CDK9, CX3CR1, TNFRSF13C, LGALS9, MX2, IFIT3, IRF7, XAF1, and IFI27 did not. Among those unique to this group, CDK9, CX3CR1, TNFRSF13C, LGALS9, IFIT3, IRF7, XAF1, and IFI27 were not previously annotated in the COVID-19 pathway. The results indicate that DEGs from our analysis likely have important roles in modulating immune responses.

### Detailed description of DEGs newly implicated to SARS-CoV-2 response

**Fig. 6:**
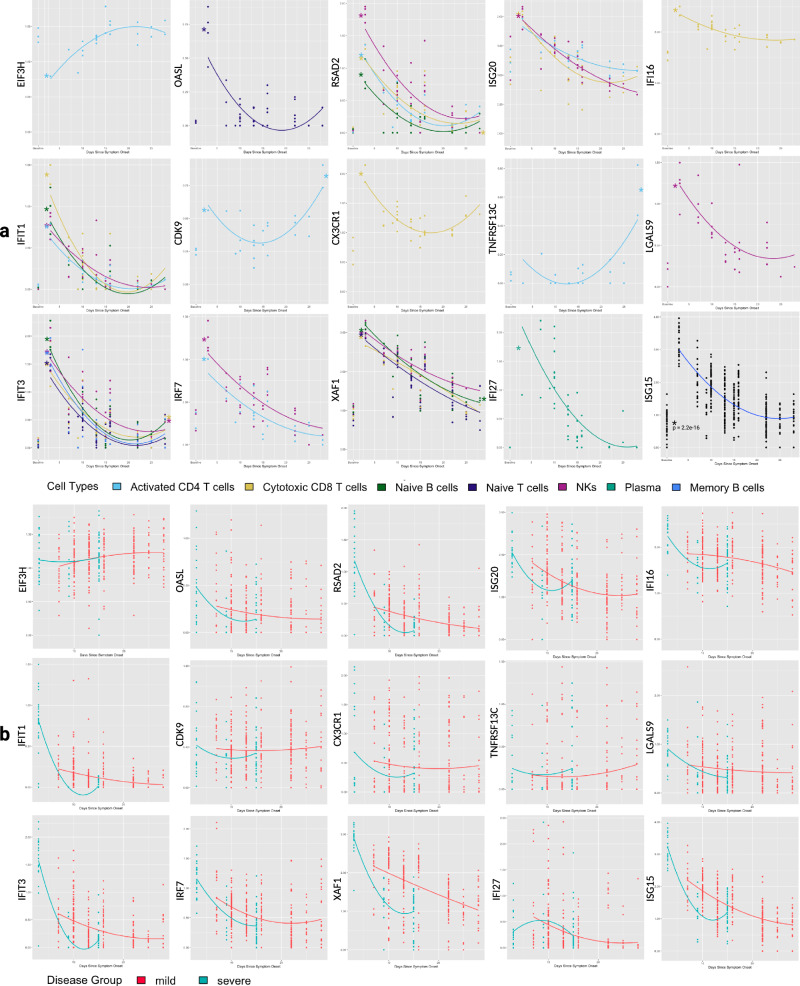
Expression vs time plots and trendlines for selected DEGs. **a** Expression through time for DEGs not previously annotated in the KEGG COVID-19 pathway. Note that for all DEGs, only corresponding cell types where they were significant are shown except *ISG15* (all sMetacell types). All baseline values were compared to day 3 (except plasma cells, which were from day 7) and day 28 via two-sided, unpaired t tests with equal variance. An asterisk is placed at the approximate average for day 3/7 and day 28 expression if significantly different (*p* < 0.05) from baseline. **b** Expression in all sMetacells through time for the DEGs in **a**, colored by disease severity. Red corresponds to mild infection while blue indicates severe.

As these DEGs changed expression post-infection, we wondered if their day 3 expression would be significantly different between infected PBMCs and controls and whether at day 28 their expression would return to the baseline. To illustrate this, we plotted the expression of DEGs associated with one of three significant GO biological process terms but not in the KEGG COVID-19 pathways, i.e. genes that are not yet well described in COVID-19 literature (Fig. 5). We compared the expression at day 3 (day 7 for plasma cells) between SARS-CoV-2 infected cells and healthy controls and found that 13/14 of these DEGs exhibited a significant difference (*p* < 0.05; two-sided t-test). Activated CD4 T was the only cell type not showing a significant difference between day 3 and baseline for *TNFRSF13C* and *ISG20*. We performed the same test between infected cells at day 28 and baseline and only found significant differences for 5 genes in certain cell types: higher for *CDK9* (in activated CD4 T cells), *IFIT3* (in NKs and Cytotoxic CD8 T cells), *XAF1* (in Naïve B cells), and *TNFRSF13C* (in activated CD4 T cells) but lower for *RSAD2* (in cytotoxic CD8 T cells). No significant difference was found for other genes, indicating the return of their expression to baseline after 28 days. Expression trends for all genes and their comparison to baseline are shown in Fig. 6. The numbers of sMetacells used for t-tests can be found in Supplementary Table 2.

We additionally investigated how the proportions of cell types in the PBMCs changed after infection but did not find good and meaningful trends, and furthermore there did not appear to be a relationship between the trend of sMetacell type proportions through time and DEG trends (Supplementary Figure 5).

*EIF3H*, whose protein product is related to cytoplasmic translation, showed increasing expression with decreasing expression velocity in activated CD4 T cells. Expression differed significantly between day 3 and baseline, which closely resembled day 28 expression. Among genes whose protein products are related to negative regulation of viral replication, *OASL* decreased expression through time with decreasing velocity in activated CD4 T cells. *RSAD2* showed the same trend in NK cells, cytotoxic CD8 T cells, activated CD4 T cells, and naïve B cells. *ISG20* decreased expression with constant velocity in activated CD4 T cells and NKs but decreased expression with decreasing velocity in cytotoxic CD8 T cells. *IFI16* expression decreased with decreasing velocity in Cytotoxic CD8 T cells while *IFIT1* expression showed the same trend in activated CD4 T cells, cytotoxic CD8 T cells, naïve B cells, and NKs. Among genes whose protein products are unique to cytokine response, *CDK9* exhibited a minima trend in activated CD4 T cells and day 28 expression was higher than baseline. *CX3CR1* expression decreased with decreasing velocity in cytotoxic CD8 T cells while *TNFRSF13C* expression increased with increasing velocity in activated CD4 T cells. *TNFRSF13C* expression at day 28 did not return to baseline. *LGALS9* (in NKs), *IFIT3* (in memory B, Naïve T, Naïve B, NKs, and cytotoxic CD8 T cells), *IRF7* (in NKs and activated CD4 T cells), and *IFI27* (in plasma cells) all showed decreasing expression with decreasing velocity and a return toward baseline expression. *XAF1* showed the same trend, but it is important to note that this gene was found to be upregulated in T, B, NKs, and DCs by Zhu et al. ^10^, which is consistent with the significant difference we observed between baseline and day 3 expression among naïve B, NKs, naïve T, and cytotoxic CD8 T cells. We further showed that *XAF1* expression decreased in these cell types through time and approached baseline (except in DCs due to our elimination of these cells from analysis).

Prior to metacell analysis by cell type, we also performed the same regression-based time series analysis on all sMetacells (irrespective of cell type) together. With the same R^2^ cutoff of > 0.5 and FDR < 0.05, we obtained one significant gene, *ISG15*. The ANOVA p-value for this gene was 8.7e-62 while the R^2^ was 0.55. There was a significant difference between baseline expression and day 3 (p = 2.2e-16).

### Correlating disease severity with the expression trends of the DEGs

Finally, we studied whether the expression trends of some DEGs were related to disease severity, again focusing on DEGs not in the KEGG COVID-19 pathway (Fig. 6b). We plotted the expression of these genes in all sMetacells through time and colored the sMetacells by whether they were from severe or mild COV-19 patients. It is interesting to note that Zhu et al. found previously that *ISG15* and *XAF1* were highly expressed in the severe COVID-19 patient at early time points then decreased through time. However, their analysis was done by grouping samples with days of PBMC collection (day 1, 3, and 16) rather than day since symptom onset. Using sMetacells and expanding the temporal resolution of this time series analysis, we showed that the high expression of these genes in the severe COVID-19 patient seemed to reflect the fact that the first sample for this patient was collected 3 days after symptom onset while the first collection for mild patients was at day 7. In addition to this, we observed that these genes and nine others (*OASL, RSAD2, ISG20, IFI16, IFIT1, CX3CR1, IFIT3, IRF7*, and *IFI27*) exhibited more rapidly declining expression in COVID-19 severe sMetacells and reached plateaus below the expression in the sMetacells from patients with mild disease. 7/11 of these genes (*RSAD2, ISG20, IFI16, IFIT1, IFIT3*, IRF7, and IFI27) are directly involved in interferon response while *OASL* is related to interleukin 27 response, which behaves similarly to interferons in its antiviral capabilities^26^. The finding of rapid decrease and lower expression of these genes at later time points in the severe COVID-19 patient is consistent with previous studies on impaired interferon response in people with severe outcomes following SARS-CoV-2 infection^27,28^.

## Discussion

### SEACells algorithm generates metacells providing statistical robustness for low replicate time series analysis

In this study, we demonstrate that metacells from the SEACells algorithm (sMetacells) can be used as replicates for time series analysis. Applying it to COVID-19 scRNA-seq data, we were able to obtain metacells that retained cell-type heterogeneity through time that appear to capture biological variances among individual patients. Despite a similar number of replicates and total cells assigned to metacells, metareplicates from the SEACells algorithm seem less prone to overfitting than those from the rMetacell method, suggesting that the retention of cell type heterogeneity could be important for decreasing overfitting when performing regression on scRNA-seq time series data. sMetacells also maintained a high degree of cell-type purity, enabling us to study expression trends for individual PBMC cell types. As such, our result suggests that this method provides a way to increase statistical power when performing quadratic regression that would otherwise be impossible due to too few replicates. In the absence of this method, pseudobulking led to overfitting, a problem thoroughly defined by Xue Ying^29^, which yielded a low number of DEGs with little biological insight. We did not systematically compare metacells from other algorithms because the SEACells paper has already demonstrated its outperformance over other software^9^. With sMetacells, we were able to obtain a list of significant DEGs for PBMC cell types through time with biological relevance to SARS-CoV-2 infection. Activated CD4 T cells contained the greatest number of significant DEGs, further validating the reliability of using the SEACells algorithm for time series analysis given CD4 T cells’ critical involvement in response to SARS-CoV-2 infection^30–33^.

### ISG15 expression changes significantly through time in PBMCs

When all PBMC sMetacells were analyzed without considering cell type information, we found that *ISG15* was the only gene showing a significant decrease in expression through time. It also exhibited decreasing expression velocity through the 28^th^ day after symptom onset. ISG15 is one of many ISGs that respond to IFN-I to establish an antiviral response^34^ and exacerbates inflammation following release from macrophages infected with SARS-CoV-2^35,36^. The combination of these findings and this gene’s significance in our analysis further establish ISG15 as an important part of the immune system’s response to SARS-CoV-2. We showed that, following infection, *ISG15* expression was initially high 3 days after symptom onset then decreased through day 28 of symptoms. Gene expression velocity also decreased, as was evidenced by the decreasing slope of the line tangent to the fitted expression curve (its derivative) through time. This makes sense since a higher degree of inflammation occurs early in infection when viral load is high then decreases as SARS-CoV-2 is cleared^37^.

In the SEACells paper, the authors found that *ISG15* expression was upregulated in CD4 T cells through approximately 10 days after symptom onset and increased again at approximately day 13. By contrast, we found that *ISG15* expression in CD4 T cells decreased continuously with decreasing velocity through approximately 25 days before returning to baseline. This difference could be due to patient cohorts or technical reasons. The SEACells authors constructed metacells from cells of all time points and then determined pseudotime of a metacell based on relative abundance of cells comprising certain time points, and their day 13 metacell was enriched in severe COVID-19 patient cells^9^. We constructed metacells using cells in each of the 10 time points separately. The difference between our results and theirs in relation to *ISG15* may be attributable to *ISG15* expression in severe COVID-19 patients. Nevertheless, because of its association with inflammation and disease severity, it will be interesting to study in the future whether changes in expression velocity of *ISG15* would lead to differences in disease severity. Although we showed a more rapid decrease in *ISG15* expression in severe COVID-19 patients, we cannot tell if the change causes severity or is in response to it. It will also be valuable to determine whether *ISG15* expression differs between those with and without long COVID-19 symptoms.

### Metacell time series analysis implies that PBMCs and type II pneumocytes share similar SARS-CoV-2 response pathways

Among 169 of the genes that we characterized with significant changes in expression through time, the protein products of 93 formed two main clusters in an interaction network generated with STRING. Within these clusters, one gene related to cytoplasmic translation, five related to negative regulation of viral genome replication, and eight related to cytokine response were already annotated in the KEGG COVID-19 pathway. Although this KEGG pathway outlines type II pneumocyte response to SARS-CoV-2 and downstream effector cell activation, its significant overlap with our DEGs suggests that despite being non-susceptible to SARS-CoV2 infection^10,38^, PBMCs may undergo a similar response to the virus as type II pneumocytes. PBMCs have been found to induce transcription of interferon-stimulated genes, such as *ISG15* mentioned above, via JAK/STAT signaling upon exposure to SARS-CoV-2^38^. The KEGG COVID-19 pathway has multiple JAK/STAT signaling cascades that are induced by various cytokines^39^. It may be the case that these same pathways are activated in PBMC response to global cytokine release upon initial infection with SARS-CoV-2.

### Metacell time series analysis implicates new genes not well described in COVID-19 literature

Among the genes not in the KEGG annotated COVID-19 pathway, all have been discussed, albeit most of them only briefly, in previously published COVID-19-related literature. For genes whose protein products are related to translation, EIF3H protein levels were previously found to be higher in human umbilical vein endothelial cells exposed to SARS-CoV-2 in vitro^40^. We found that *EIF3H* expression increased through time with decreasing expression velocity in activated CD4 T cells. Interestingly, earlier time points showed lower expression compared to baseline, which deviates slightly from Melo et al.’s findings. However, the increasing expression trend of this gene suggests that CD4 T cells may play an important role in SARS-CoV-2 translation inhibition.

For genes whose protein products are related to interferon response, *IFI27* expression in blood was found to be more highly expressed in patients infected with SARS-CoV-2 as determined via qPCR^41^. Our results show that *IFI27* expression decreases significantly through time with decreasing expression velocity before returning to baseline in plasma cells. This suggests that plasma cells may be a large contributor to high *IFI27* expression in COVID-19 patient blood. *IFIT3* was found to increase in expression through time in SARS-CoV-2 infected mice through 8 days of infection^42^. Interestingly, this conflicts with our results, which showed that *IFIT3* expression decreased through time with decreasing expression velocity in naïve T cells, naïve B cells, memory B cells, NKs, and cytotoxic CD8 T cells. A previous study showed that among interferon-stimulated genes, *IFIT3, ISG15, IFIT2, ISG20, IRF7*, and *MX2* were downregulated in monocytes^43^. All these genes except *IFIT2* were among genes that we deemed to as changing significantly through time in a variety of cell types, all with decreasing trends. We found that *IFIT3, ISG15, ISG20*, and *IRF7* were actually upregulated at day 3 compared to baseline before decreasing back to baseline. Perhaps if the time course were extended, we would see a more global decrease below baseline for these genes. However, more consistent with Maher et al., we found that these genes showed lower expression in the severe COVID-19 patients, further suggesting that downregulation of these interferon response genes may confer greater susceptibility to infection. We question whether this trend, along with expression velocity, differs depending on previous exposure to SARS-CoV-2 or other coronaviruses. We also noticed that *IRF7* and *ISG20* expression fell slightly below baseline near 28 days postsymptom-onset, suggesting potential downregulation of these genes at later stages of infection.

Among genes whose protein products are related to cytokine response, we found that *XAF1* expression decreased with decreasing velocity in NKs, naïve B, naïve T, and CD8 T cells. Its expression was significantly greater at day 3 compared to baseline, and by day 28, expression was still slightly above baseline levels (only significant for Naïve B cells). Several studies have outlined *XAF1*’s role in protecting against RNA virus infection^44,45^, so high expression at early time points makes sense. However, as mentioned previously, this gene’s expression decreased more rapidly in the patient with severe COVID-19. This may suggest that its downregulation increases risk of developing severe COVID-19 due to diminished antiviral activity. We also found that *TNFRSF13C* expression increased in CD4 T cells through 28 days. One group found that a gain of function mutation in this gene was associated with severe COVID-19^46^. We did not find that this gene was more highly expressed in the severe COVID-19 patient; however, this patient did not have a collection at 28 days. *CX3CR1* expression in NKs has been associated with severe COVID-19^47^. Our data showed a significant change in expression through time for this gene in cytotoxic CD8 T cells. *CX3CR1* expression decreased with decreasing velocity; however, there was also a slight increase in expression after day 20. Day 28 expression was higher than baseline, but this difference was not significant. Given *CX3CR1*’s association with severe disease and the role of chemokines in inflammation^48^, we suggest that this gene may contribute to long COVID-19 symptoms if it continues to be expressed above baseline following virus clearance. Future studies should therefore determine expression trends through time for *CX3CR1* in patients with long COVID-19 compared to patients who fully recover.

Although several other significant DEGs from our analysis have been discussed in literature related to COVID-19, our data did not further contribute to understanding their potential roles in SARS-CoV-2 infection. We comment here only on those where our results are most contributory to previously published materials.

### Metacells uncover important differences in expression trends by disease severity

Using sMetacells, we found 11 DEGs exhibiting an expression trend in severe COVID-19 sMetacells that was initially high then decreased below that of mild COVID-19 after one week of symptoms. These genes are *ISG15, XAF1, OASL, RSAD2, ISG20, IFI16, IFIT1, CX3CR1, IFIT3, IRF7*, and IFI27. Zhu et al. found that *ISG15* and *XAF1* were upregulated in the severe COVID-19 patient. However, their finding may be explained by how samples were collected differently between the severe and mild group and the higher expression of these genes at the early stage (Fig. 6). Different from their findings, we suggest that these genes, along with *OASL, RSAD2, ISG20, IFI16, IFIT1, CX3CR1, IFIT3, IRF7*, and IFI27, had rapidly decreased expression in the severe COVID-19 patients and lower expression than in the mild disease patients. Importantly, *ISG15, RSAD2, ISG20, IFI16, IFIT1, IFIT3, IRF7*, and *IFI27* are all related to interferon response. Their diminished expression at later time points is consistent with previous studies regarding interferon response in patients infected with SARS-CoV-2. One group found that type I interferon response was impaired in severe COVID-19^28^, while another described how immunodeficiencies reducing type I interferon response confer a high risk for critical COVID-19^49^. IFIT1 was found to inhibit EIF3^50^, which functions in translation initiation. If our results represent a true decrease in IFIT1 levels through time, they would be consistent with increasing *EIF3* expression through time **(**Fig. 6a).

Interestingly a recent longitudinal study using ~300 COVID-19 patient samples reported similar trends for interferon response^51^. They found that the upregulation of 13 genes (*ISG15, ISG20, IFIT1, IFIT2, IFIT3, IFITM1, IFI6, IFI35, BST2, RSAD2, IRF7, MX1* and *MX2*) was the signature of early gene expression for patients to subsequently develop worse symptoms. Additionally, they found that these genes decreased rapidly in the severe patients, in accordance with our finding using a much smaller number of patient samples (*n* = 5), but we only found about half of their genes (*ISG15*, *ISG20, IFIT1, IFIT3, RSAD2*, and *IRF7*). Although it could be valuable to apply our metacell approach to this large cohort in the future, the agreement provides a strong support for using sMetacells to analyze time series data with a few replicates.

### Limitations

Our study is a proof of concept and generally needs to be applied to more datasets. Method wise, it particularly needs to be tested more systematically with datasets containing more biological replicates to carefully study the performance difference between true biological replicates and metareplicates. In terms of the relationship of our results to COVID-19, the patient numbers were quite small, and especially our comparison of day 28 expression to baseline is suboptimal given the low number of metacells per cell type at day 28. Our analysis of expression trends by cell type was also limited by the overall low cell count for certain cell types. This led to low numbers of metacells and subsequent overfitting for these cell types.

## Methods

### Metacell Creation

The COVID-19 scRNA-seq dataset was obtained from a previous study that performed time series analysis on PBMCs from five SARS-CoV-2 infected patients^10^. The authors normalized the data using the Seurat software’s^52^ “NormalizeData” function where gene counts for a cell are divided by total counts for that cell and then natural log transformed. They further integrated data from different samples using the “FindIntegrationAnchors” and “IntegrateData” functions, prior to dimensionality reduction and clustering. We used the authors’ processed data directly. The date of symptom onset and sample collection were recorded for each patient. Since we did not intend to group patients by disease stages, we simply classified each collected sample by the number of days after symptom onset. Samples from influenza-infected patients were excluded from our analysis, as were controls, since they were not collected continuously through time. However, we included the normalized expression of three healthy controls as baseline values for comparative purposes.

For SEACells, the number of metacells was determined based on the software authors’ suggestion of 1 metacell per 75 single cells^9^. We rounded to the nearest 10 to enable the creation of more metacells for time points with fewer total cells. We created twenty (rather than ten) day 28 metacells for better comparison to baseline values. We used the SEACells (version 0.3.3) algorithm implemented in Python 3.9. We applied it to samples of each time point independently. For each of the 10-time points, the input consisted of an Anndata object containing pre-normalized gene counts, the n most highly variable genes (2000 for our dataset), cell cluster/type assignments, and a low dimensional representation of the data, all established by the original authors^10^. Subsequent steps for metacell creation were outlined in the SEACells manuscript^9^ and in Fig. 1. The expression of each gene for a given metacell was determined by averaging the normalized counts of the cells that were assigned to it (Fig. 1a, b). Each metacell was ascribed a cell type based on whichever cell type was most prominent among the assigned individual cells. For example, if most cells assigned to a metacell were plasma cells, the metacell would be called a plasma metacell. The percentage of cells comprising the metacell that were of the assigned cell type was referred to as its “purity.” Here, we called metacells created using the SEACells algorithm “sMetacells.”

To obtain metacells composed of random individual cells by cell type, which we called “rMetacells”, we subset the same filtered PBMC dataset by time. We then subset by cell type and took the average normalized expression of 20 randomly selected cells to create an rMetacell. While we intended to use more cells to create metacells that were as comparable to sMetacells as possible, several cell types had less than 75 cells for a particular time point, so we decreased our threshold to maximize metacell assignments. The SEACells algorithm was not confined to this issue due to its ability to assign varying numbers of cells to each metacell based on nearest neighbor determination.

The sMetacells and rMetacells thus differ in two major ways (Fig. 1): 1) rMetacells were created at random and therefore more likely to represent the densest space of each cluster, whereas sMetacells accounted for this, making them more heterogenous; and 2) rMetacells had exactly 20 cells assigned to them, all from the same cell type, whereas sMetacells had a varying number of cells assigned, which were not confined to a certain cell type, further contributing to their diversity.

### maSigPro and Trend Determination

After the creation of metacells (by SEACells or randomly) for each time point, maSigPro (version 1.72) was used to find DEGs through time. The maSigPro approach utilizes regression ANOVA followed by a variable selection procedure^25^. Quadratic regression was used since we expected the change in gene expression to follow one of eight general trends, as described in Fig. 1c, d. For each cell type and gene, a quadratic equation was generated to represent expression through time. Only genes with an ANOVA FDR (false discovery rate) corrected *p* value (determined from the model’s F-statistic) < 0.05 and R^2^ value > 0.5 were considered as DEGs. The null hypothesis for the F-test is that the coefficients generated for fitted line are zero. We included R^2^ to account for how well the model predicted observed outcomes. The stepwise regression step calculated a p-value for each coefficient A, B, and C in Eq. 1, which were used for expression trend determination, where the quadratic term (coefficient A) dictates the shape of the fitted line.

1 $$y=A{x}^{2}+{Bx}+C$$

The p-values for the quadratic terms were used to determine whether the line was linear or parabolic. If *p* < 0.05 and the absolute value of the slope of the line tangent to the expression vs time curve (the expression velocity) decreased through time, we called this decreasing velocity, denoted “↓ velocity” in figures. If *p* < 0.05 and the absolute value of the slope of the tangent line increased, this was referred to as increasing velocity, or “↑ velocity.” We combined these terms with the overall trend of increasing or decreasing expression. For example, if the expression of a gene was decreasing through time, was not linear, and showed decreasing velocity, we would call this decreasing expression with decreasing expression velocity or “Decreasing, ↓ velocity” for short. If *p* > 0.05 for the quadratic term, we considered this to be linear and the direction of the curve dictated whether it was considered increasing or decreasing. Increasing linear expression is synonymous with “Increasing, constant” while decreasing linear expression is synonymous with “Decreasing, constant” where constant refers to the expression velocity. If the average expression for the first time point and the last time point were both less than each of the time points between them, this was considered “Maxima”. If greater, this was “Minima”.

We should note that DEGs from dendritic cells (DCs), megakaryocytes, monocytes, cycling plasma, and stem cells were eliminated from further analysis due to low cell numbers (less than 500 in total across all time points), which led to numbers of metacells too low for robust statistical analysis, because performing quadratic regression would lead to overfitting for these cell types. Additionally, due to low metacell counts for the first three time points in memory B cells, we eliminated days 3, 7, and 9 metacells for trend determination of this cell type due to skewing toward early time point outliers. For all other cell types, all 10-time points (days 3, 7, 9, 10, 13, 15, 16, 22, 25, and 28) were included for trend determination.

### Other Bioinformatics Databases and Tools

For classification of the functions of the gene products (i.e., proteins), we used the DAVID Gene Function Annotation Tool^53,54^ and further grouped selected terms into broader function categories, such as transferases, protein degradation, immunoglobulin-related, and immune-related. The KEGG^39^ COVID-19 pathway was used to define known SARS-CoV-2-related genes. Although the KEGG pathway is based on SARS-CoV-2 entry into type 2 pneumocytes, we generalized this response to the cascade of events that follow the uptake of the virus by PBMCs to further narrow our search for novel expression responses. We base this generalization on the finding that cell-intrinsic innate immune responses are triggered in PBMCs following exposure to SARS-CoV-2^38^. The STRING Database^55^ was used for network analysis to connect our DEGs to known COVID-19-related genes. To find significantly enriched gene ontology (GO) terms from inputted DEGs, we used geneontology.org^56,57^, set the annotation dataset to “PANTHER GO-slim biological processes”, and used the entire human genome as background. Figures were edited using biorender.com.

### Reporting summary

Further information on research design is available in the Nature Research Reporting Summary linked to this article.

### Supplementary information

Supplementary Figures

Supplementary Tables

Reporting summary

## Data Availability

The scRNA-seq data reanalyzed in the current study were described in a previous study (ref. 10) and publicly available at the CNGB Nucleotide Sequence Archive (accession number: CNP0001102).

## Code availability

The R codes used for the data analysis are available at the GitHub, https://github.com/bioinfoDZ/metacell_based_DEA.

## References

[1] Heumos L (2023). Best practices for single-cell analysis across modalities. Nat. Rev. Genet..

[2] Stuart T, Satija R (2019). Integrative single-cell analysis. Nat. Rev. Genet..

[3] Squair JW (2021). Confronting false discoveries in single-cell differential expression. Nat. Commun..

[4] Zhang X (2019). Comparative Analysis of Droplet-Based Ultra-High-Throughput Single-Cell RNA-Seq Systems. Mol. Cell.

[5] Ziegenhain C (2017). Comparative Analysis of Single-Cell RNA Sequencing Methods. Mol. Cell.

[6] Baran, Y. et al. MetaCell: analysis of single-cell RNA-seq data using K-nn graph partitions. (1474-760X (Electronic)). 10.1186/s13059-019-1812-2 PMC6790056 31604482

[7] Ben-Kiki, O. et al. Metacell-2: a divide-and-conquer metacell algorithm for scalable scRNA-seq analysis. (1474-760X (Electronic)). 10.1186/s13059-022-02667-1 PMC9019975 35440087

[8] Bilous, M. et al. Metacells untangle large and complex single-cell transcriptome networks. (1471-2105 (Electronic)). 10.1186/s12859-022-04861-1 PMC9375201 35963997

[9] Persad, S, et al. SEACells infers transcriptional and epigenomic cellular states from single-cell genomics data. Nature Biotechnology, 2023. 10.1038/s41587-023-01716-9 PMC10713451 36973557

[10] Zhu L (2020). Single-Cell Sequencing of Peripheral Mononuclear Cells Reveals Distinct Immune Response Landscapes of COVID-19 and Influenza Patients. Immunity.

[11] Gorbalenya AE (2020). The species Severe acute respiratory syndrome-related coronavirus: classifying 2019-nCoV and naming it SARS-CoV-2. Nat. Microbiol..

[12] Wang C (2020). A novel coronavirus outbreak of global health concern. Lancet.

[13] WHO COVID-19 Dashboard. 2020, Geneva: World Health Organization.

[14] Lotfi, M., Hamblin, M. R. & Rezaei, N. COVID-19: Transmission, prevention, and potential therapeutic opportunities. (1873-3492 (Electronic)). 10.1016/j.cca.2020.05.044 PMC7256510 32474009

[15] Yuan J (2020). Monitoring transmissibility and mortality of COVID-19 in Europe. Int. J. Infect. Dis..

[16] Gallo Marin B (2021). Predictors of COVID‐19 severity: A literature review. Rev. Med. Virol..

[17] Teixeira da Silva JA, Tsigaris P, Erfanmanesh M (2021). Publishing volumes in major databases related to Covid-19. Scientometrics.

[18] Yang Y (2023). Paperdemic’ during the COVID-19 pandemic. Eur. J. Intern. Med..

[19] Kleiveland, C. R. *Peripheral Blood Mononuclear Cells.* 161–167 (Springer International Publishing, 2015). 29787062

[20] Bergamaschi, L. et al. Longitudinal analysis reveals that delayed bystander CD8+ T cell activation and early immune pathology distinguish severe COVID-19 from mild disease. (1097–4180 (Electronic)). 10.1016/j.immuni.2021.05.010 PMC8125900 34051148

[21] Stephenson E (2021). Single-cell multi-omics analysis of the immune response in COVID-19. Nat. Med..

[22] Huo L (2021). Single-cell multi-omics sequencing: application trends, COVID-19, data analysis issues and prospects. Brief. Bioinforma..

[23] Wang X (2022). Temporal transcriptomic analysis using TrendCatcher identifies early and persistent neutrophil activation in severe COVID-19. JCI Insight.

[24] Liu C (2021). Time-resolved systems immunology reveals a late juncture linked to fatal COVID-19. Cell.

[25] Nueda, M. J., S. Tarazona, S. & Conesa, A. Next maSigPro: updating maSigPro bioconductor package for RNA-seq time series. (1367-4811 (Electronic)). 10.1093/bioinformatics/btu333 PMC4155246 24894503

[26] Amsden, H. et al. Antiviral Activities of Interleukin-27: A Partner for Interferons? (1664-3224 (Electronic)). 10.3389/fimmu.2022.902853 PMC9134790 35634328

[27] Eskandarian Boroujeni, M. et al. Dysregulated Interferon Response and Immune Hyperactivation in Severe COVID-19: Targeting STATs as a Novel Therapeutic Strategy. (1664–3224 (Electronic)). 10.3389/fimmu.2022.888897 PMC9156796 35663932

[28] Hadjadj J (2020). Impaired type I interferon activity and inflammatory responses in severe COVID-19 patients. Science.

[29] Ying, X. An overview of overfitting and its solutions. in *Journal of physics: Conference series*. 2019. IOP Publishing.

[30] Cox RJ, Brokstad KA (2020). Not just antibodies: B cells and T cells mediate immunity to COVID-19. Nat. Rev. Immunol..

[31] Chen Z, John Wherry E (2020). T cell responses in patients with COVID-19. Nat. Rev. Immunol..

[32] Grifoni A (2020). Targets of T Cell Responses to SARS-CoV-2 Coronavirus in Humans with COVID-19 Disease and Unexposed Individuals. Cell.

[33] Koblischke M (2020). Dynamics of CD4 T cell and antibody responses in COVID-19 patients with different disease severity. Front. Med..

[34] Perng Y-C, Lenschow DJ (2018). ISG15 in antiviral immunity and beyond. Nat. Rev. Microbiol..

[35] Cao X (2021). ISG15 secretion exacerbates inflammation in SARS-CoV-2 infection. Nat. Immunol..

[36] Munnur D (2021). Altered ISGylation drives aberrant macrophage-dependent immune responses during SARS-CoV-2 infection. Nat. Immunol..

[37] Lariccia V (2020). Challenges and Opportunities from Targeting Inflammatory Responses to SARS-CoV-2 Infection: A Narrative Review. J. Clin. Med..

[38] Kazmierski J (2022). Nonproductive exposure of PBMCs to SARS-CoV-2 induces cell-intrinsic innate immune responses. Mol. Syst. Biol..

[39] Kanehisa M (2002). The KEGG databases at GenomeNet. Nucleic Acids Res..

[40] de Melo TC (2022). Proteomic Analysis Identifies Molecular Players and Biological Processes Specific to SARS-CoV-2 Exposure in Endothelial Cells. Int J. Mol. Sci..

[41] Shojaei M (2023). IFI27 transcription is an early predictor for COVID-19 outcomes, a multi-cohort observational study. Front. Immunol..

[42] Gao X (2021). Genome‐wide screening of SARS‐CoV‐2 infection‐related genes based on the blood leukocytes sequencing data set of patients with COVID‐19. J. Med. Virol..

[43] Maher AK (2022). Transcriptional reprogramming from innate immune functions to a pro-thrombotic signature by monocytes in COVID-19. Nature. Communications.

[44] Kuang M (2023). XAF1 promotes anti-RNA virus immune responses by regulating chromatin accessibility. Sci. Adv..

[45] Han Y (2022). XAF1 Protects Host against Emerging RNA Viruses by Stabilizing IRF1-Dependent Antiviral Immunity. J. Virol..

[46] Russo R (2021). The TNFRSF13C H159Y Variant Is Associated with Severe COVID-19: A Retrospective Study of 500 Patients from Southern Italy. Genes (Basel).

[47] Liechti T (2022). Immune phenotypes that are associated with subsequent COVID-19 severity inferred from post-recovery samples. Nat. Commun..

[48] Moser B (2004). Chemokines: role in inflammation and immune surveillance. Ann. Rheum. Dis..

[49] Zhang Q (2022). Human genetic and immunological determinants of critical COVID-19 pneumonia. Nature.

[50] Diamond MS, Farzan M (2013). The broad-spectrum antiviral functions of IFIT and IFITM proteins. Nat. Rev. Immunol..

[51] Lin QXX (2024). Longitudinal single cell atlas identifies complex temporal relationship between type I interferon response and COVID-19 severity. Nat. Commun..

[52] Hao Y (2023). Dictionary learning for integrative, multimodal and scalable single-cell analysis. Nat. Biotechnol..

[53] Sherman, B. T. et al. DAVID: a web server for functional enrichment analysis and functional annotation of gene lists (2021 update). (Electronic)). 10.1093/nar/gkac194 PMC9252805 35325185

[54] Huang da, W. R.A. Sherman Bt Fau - Lempicki, and R.A. Lempicki, *Systematic and integrative analysis of large gene lists using DAVID bioinformatics resources*. (1750-2799 (Electronic)). 10.1038/nprot.2008.211 19131956

[55] Szklarczyk, D. et al. The STRING database in 2023: protein-protein association networks and functional enrichment analyses for any sequenced genome of interest. (Electronic)). 10.1093/nar/gkac1000 PMC9825434 36370105

[56] Ashburner M (2000). Gene Ontology: tool for the unification of biology. Nat. Genet..

[57] The Gene Ontology C (2023). The Gene Ontology knowledgebase in 2023. Genetics.

