# Supplementary material for: Metacell-based differential expression analysis identifies cell type specific temporal gene response programs in COVID-19 patient PBMCs
Source: NPJ Syst Biol Appl. 2024 Apr 5;10:36. doi: 10.1038/s41540-024-00364-2 (PMC10997786; doi:10.1038/s41540-024-00364-2)
Supplement: Supplementary file 1 — Supplementary Figures [file 41540_2024_364_MOESM1_ESM.pdf]

Metacell-based differential expression analysis identifies cell type specific temporal gene response programs in COVID-19 patient PBMCs

Kevin O'Leary[1], Deyou Zheng [1,2,3*]

1. Department of Genetics, Albert Einstein College of Medicine, Bronx, NY, USA

2. Department of Neurology, Albert Einstein College of Medicine, Bronx, NY, USA

3. Department of Neuroscience, Albert Einstein College of Medicine, Bronx, NY, USA

Supplementary Materials

I. Supplementary Tables 1-3 (in a separated Excel file)

Supplementary Table 1: Summary of the average expression standard deviation across cell types. Only included data for cell types that were compared between sMetacells and rMetacells. SD was calculated for each gene at each time point for every cell type. The mean SD (mSD) across all genes was then determined and shown. Values of 0 indicate only one metareplicate for that time point and therefore no variance/SD. "Na" indicates that there were no metareplicates for that time point.

Supplementary Table 2: Summary of the numbers of patient samples, single cells, and SEACells-generated sMetacells. A, the number of samples and cells for each of the 10 time points. B, the number of sMetacells representing each cell type per time point.

Supplementary Table 3: All genes deemed statistically significant using maSigPro by cell type and used for downstream analysis. FDR adjusted p-values are all less than 0.05 while R^2 is greater than 0.5. For each gene and cell type, the overall expression trend pattern is also shown.

II. Supplementary Figures 1-5

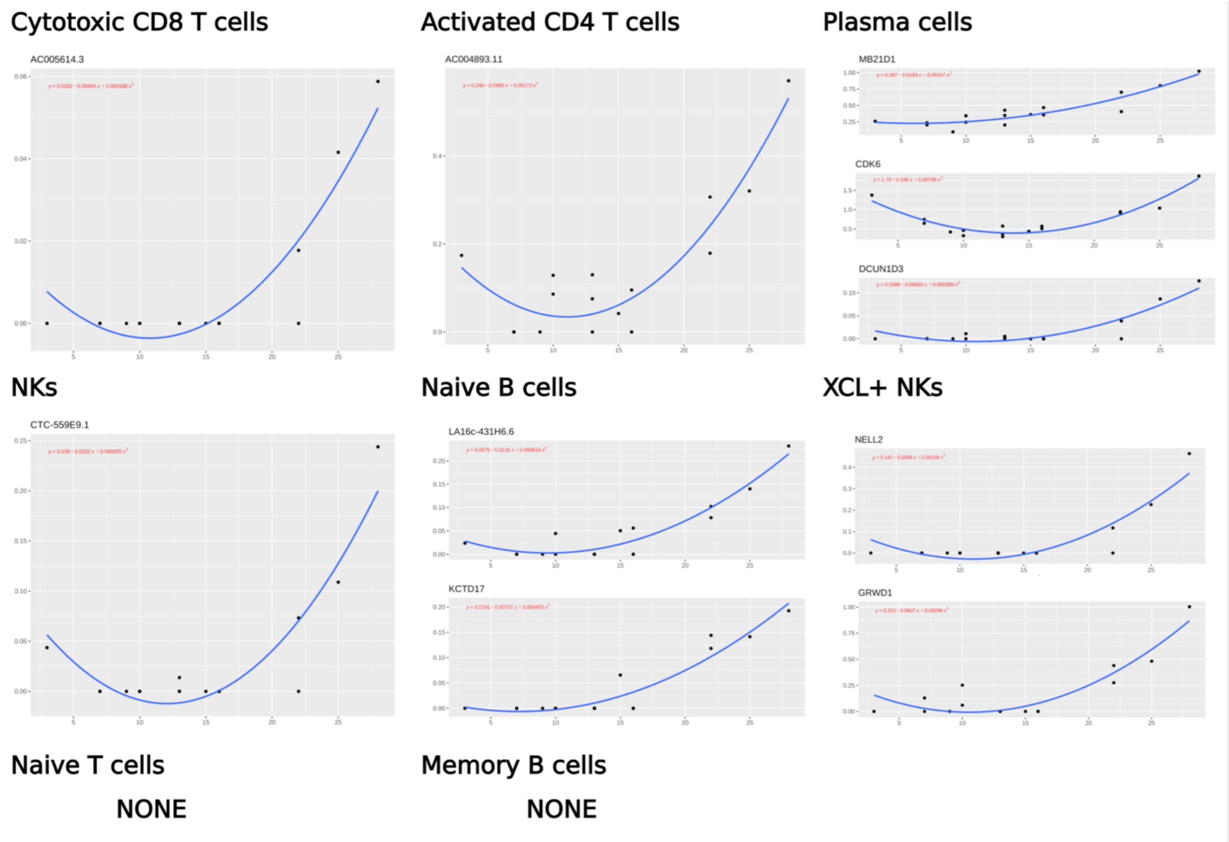

Supplementary Figure 1: Pseudobulk quadratic regression curves for significantly differentially expressed genes (FDR adjusted p < 0.05 and R^2 > 0.5) by cell types with formula. Two of the 8 cell types did not have significant genes, naïve T cells and memory B cells.

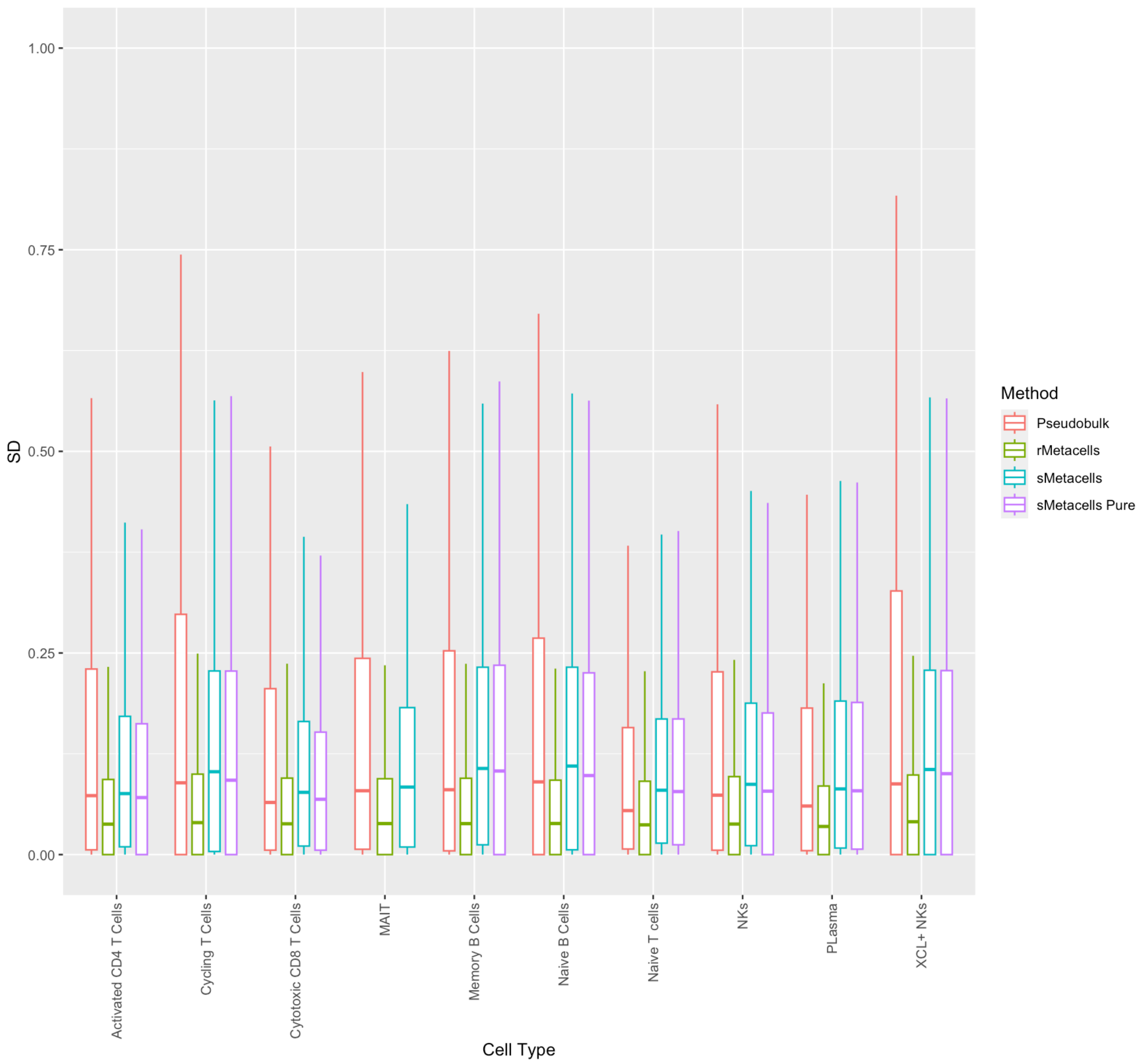

Supplementary Figure 2: Comparison of gene expression SDs for rMetacells, sMetacells, and pseudobulked samples. Data for all cell types and all time points were combined for computing these SDs. The SDs for “sMetacells Pure” were computed for the subset of sMetacells with >95% composed cells belonging to one type. The lower and upper hinges of the boxplots represent the first and third quartiles, the center line is the median, and the whiskers extend no further than 1.5 * inter quartile range.

a, naïve T cells

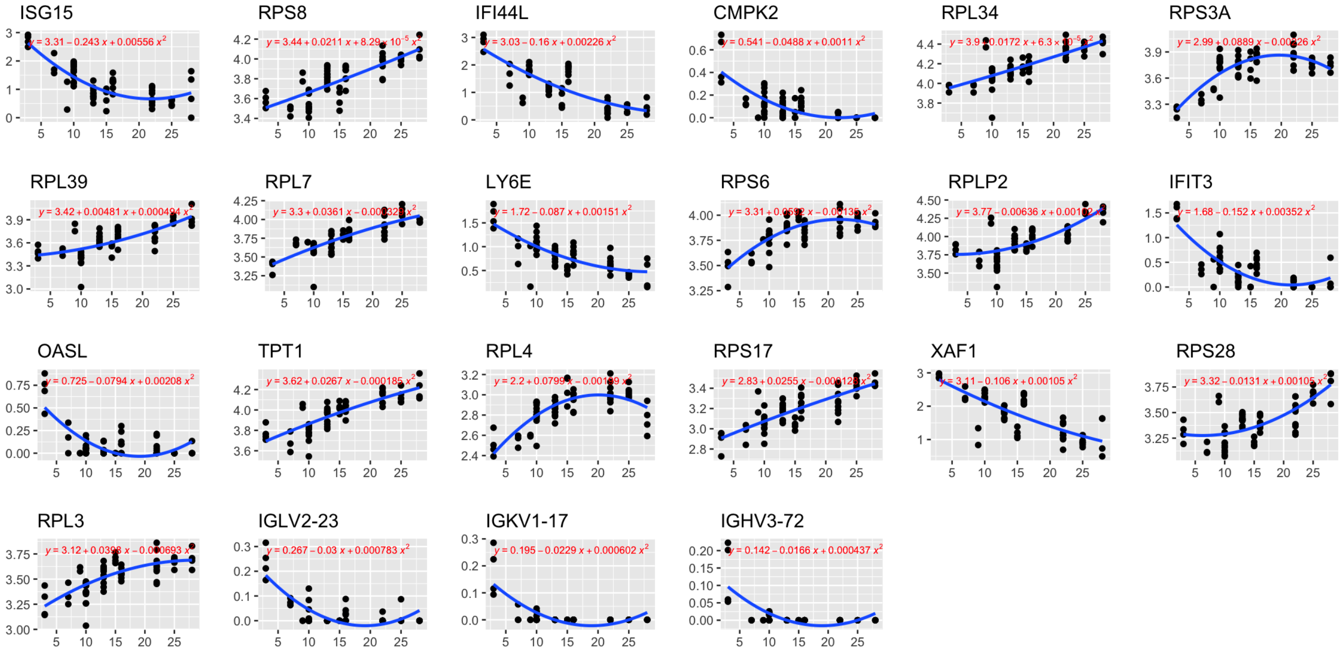

b, naïve B cells

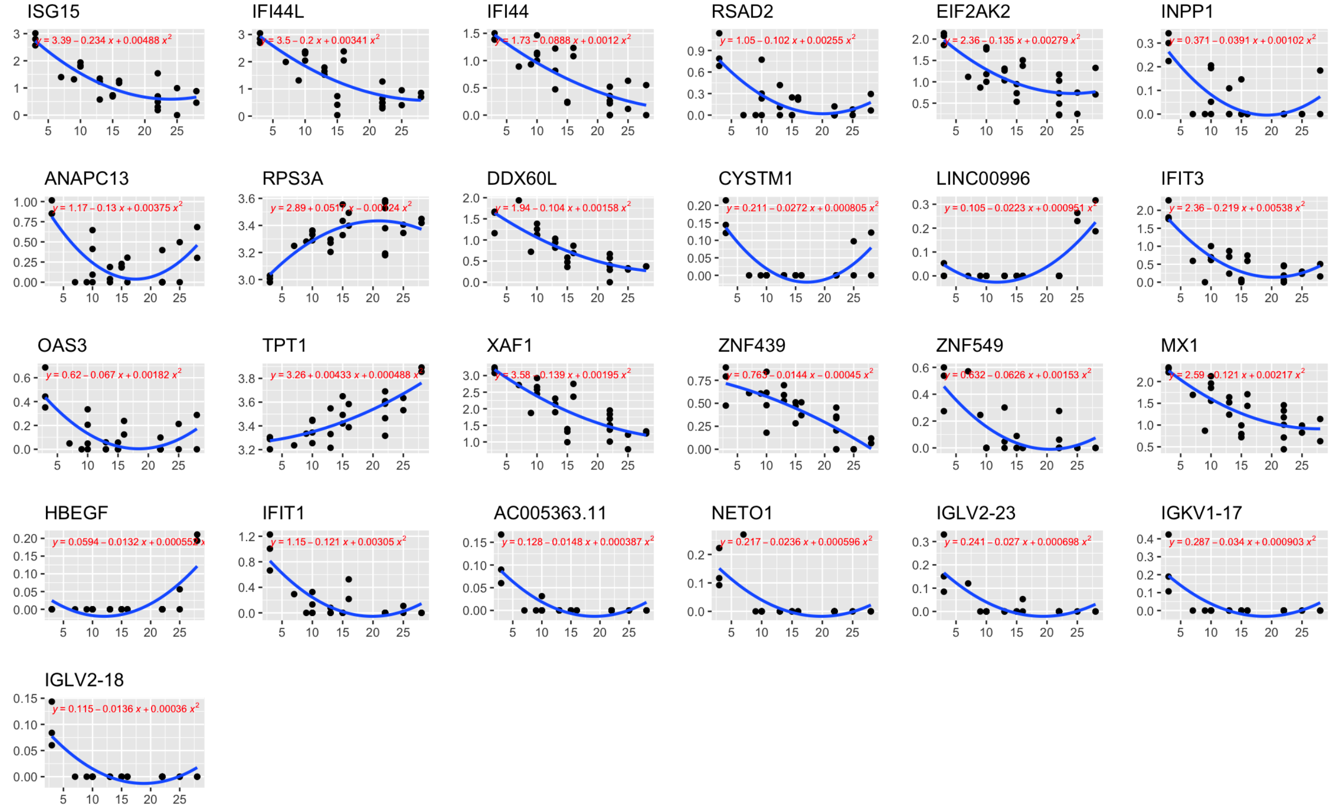

c, activated CD4 T

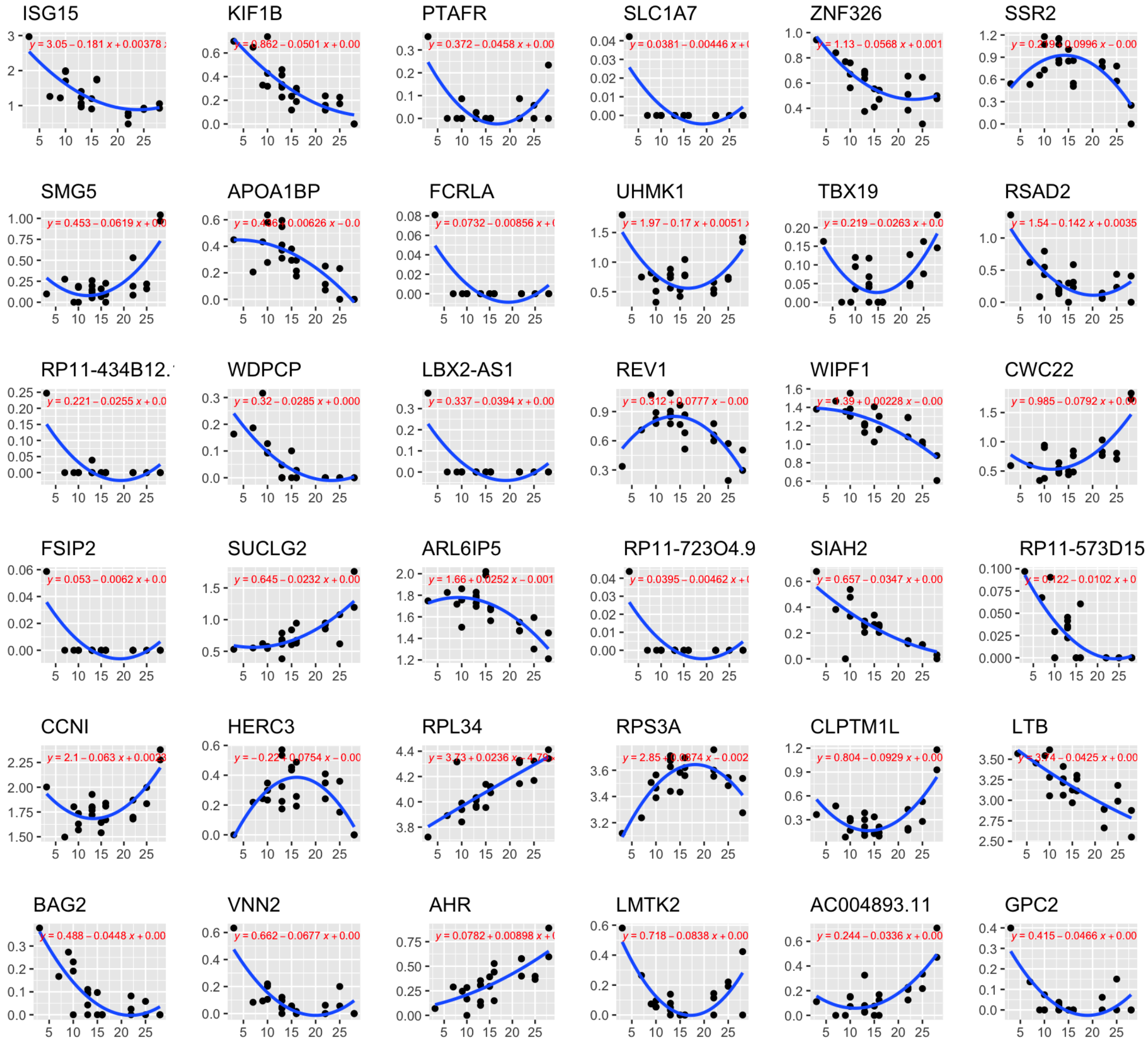

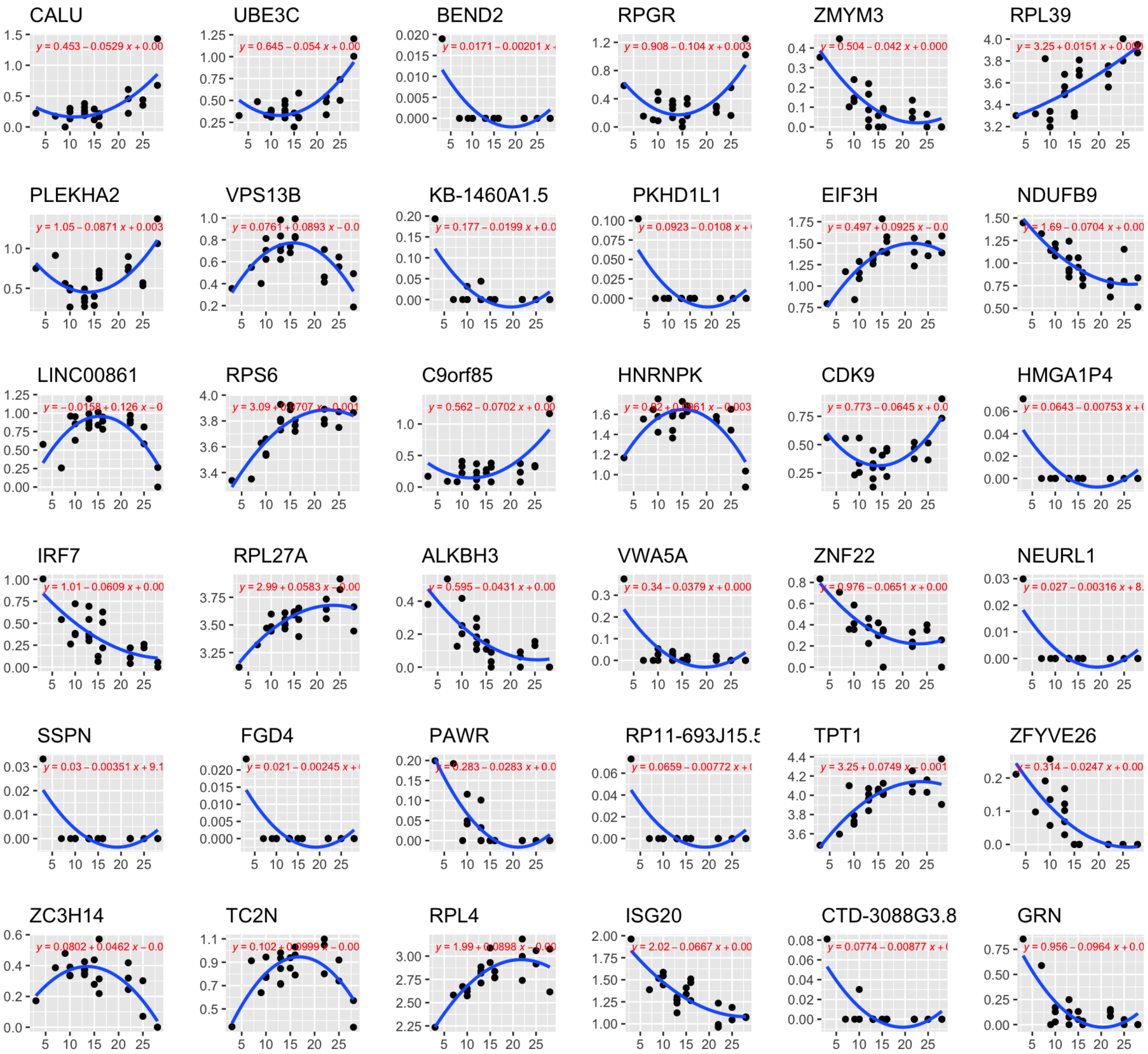
CALU
UBE3C
BEND2
RPGR
ZMYM3
RPL39
PLEKHA2
VPS13B
KB-1460A1.5
PKHD1L1
EIF3H
NDUFB9
LINC00861
RPS6
C9orf85
HNRNPK
CDK9
HMGA1P4
IRF7
RPL27A
ALKBH3
VWA5A
ZNF22
NEURL1
SSPN
FGD4
PAWR
TPT1
ZFYVE26
ZC3H14
TC2N
RPL4
ISG20
CTD-3088G3.8
GRN

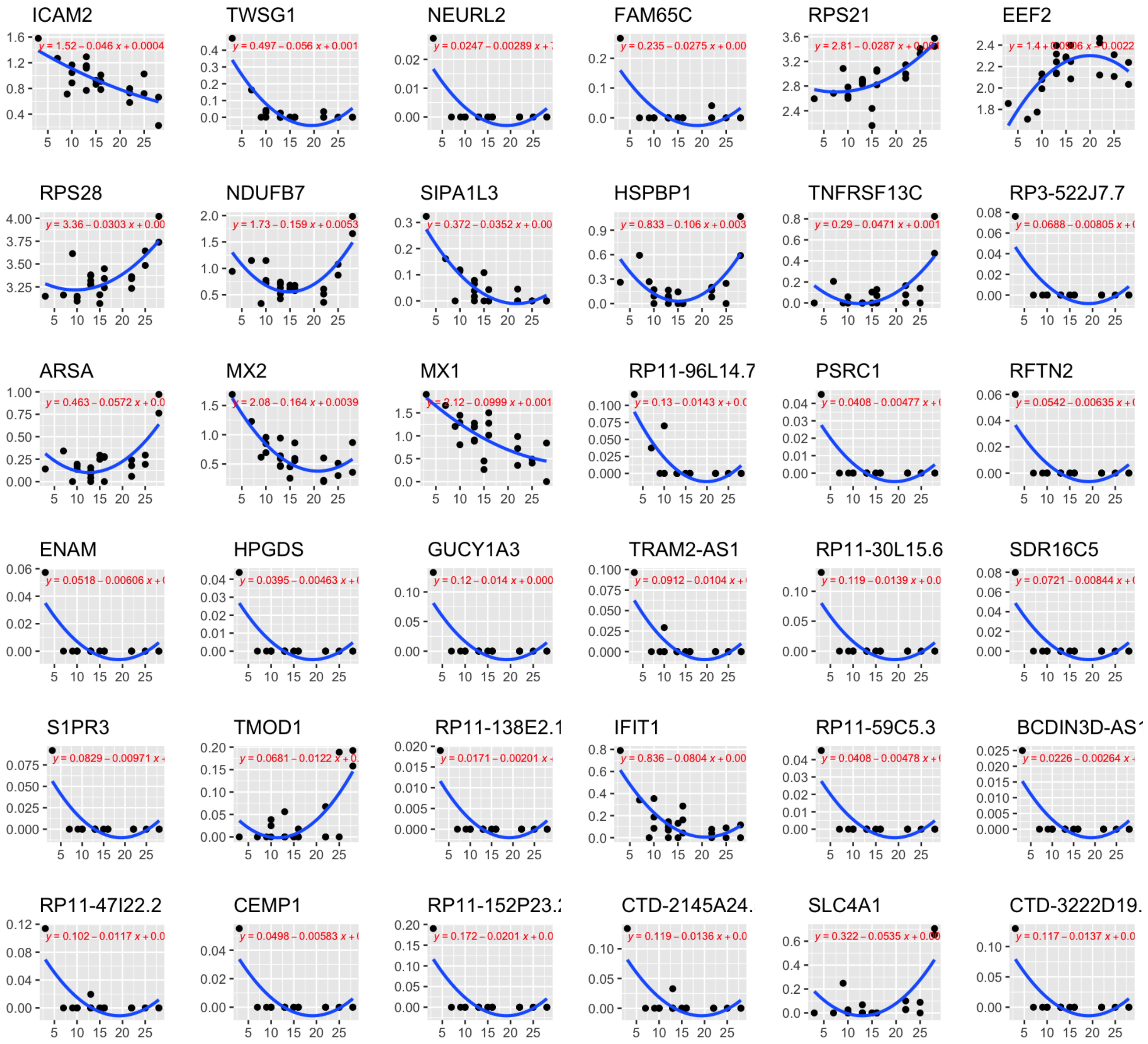
ICAM2
y = 1.52 – 0.046 x + 0.0004
TWSG1
y = 0.497 – 0.056 x + 0.001
NEURL2
y = 0.0247 – 0.00289 x +
FAM65C
y = 0.235 – 0.0275 x + 0.00
RPS21
y = 2.81 – 0.0287 x + 0.00
EEF2
y = 1.4 + 0.0906 x – 0.0022
RPS28
y = 3.36 – 0.0303 x + 0.00
NDUFB7
y = 1.73 – 0.159 x + 0.0053
SIPA1L3
y = 0.372 – 0.0352 x + 0.00
HSPBP1
y = 0.833 – 0.106 x + 0.003
TNFRSF13C
y = 0.29 – 0.0471 x + 0.001
RP3-522J7.7
y = 0.0688 – 0.00805 x +
ARSA
y = 0.463 – 0.0572 x + 0.0
MX2
y = 2.08 – 0.164 x + 0.0039
MX1
y = 2.12 – 0.0999 x + 0.001
RP11-96L14.7
y = 0.13 – 0.0143 x + 0.0
PSRC1
y = 0.0408 – 0.00477 x +
RFTN2
y = 0.0542 – 0.00635 x +
ENAM
y = 0.0518 – 0.00606 x +
HPGDS
y = 0.0395 – 0.00463 x +
GUCY1A3
y = 0.12 – 0.014 x + 0.000
TRAM2-AS1
y = 0.0912 – 0.0104 x +
RP11-30L15.6
y = 0.119 – 0.0139 x + 0.0
SDR16C5
y = 0.0721 – 0.00844 x +
S1PR3
y = 0.0829 – 0.00971 x +
TMOD1
y = 0.0681 – 0.0122 x + 0.
RP11-138E2.1
y = 0.0171 – 0.00201 x +
IFIT1
y = 0.836 – 0.0804 x + 0.00
RP11-59C5.3
y = 0.0408 – 0.00478 x +
BCDIN3D-AS1
y = 0.0226 – 0.00264 x +
RP11-47I22.2
y = 0.102 – 0.0117 x + 0.0
CEMP1
y = 0.0498 – 0.00583 x +
RP11-152P23.2
y = 0.172 – 0.0201 x + 0.0
CTD-2145A24.
y = 0.119 – 0.0136 x + 0.0
SLC4A1
y = 0.322 – 0.0535 x + 0.00
CTD-3222D19.
y = 0.117 – 0.0137 x + 0.0

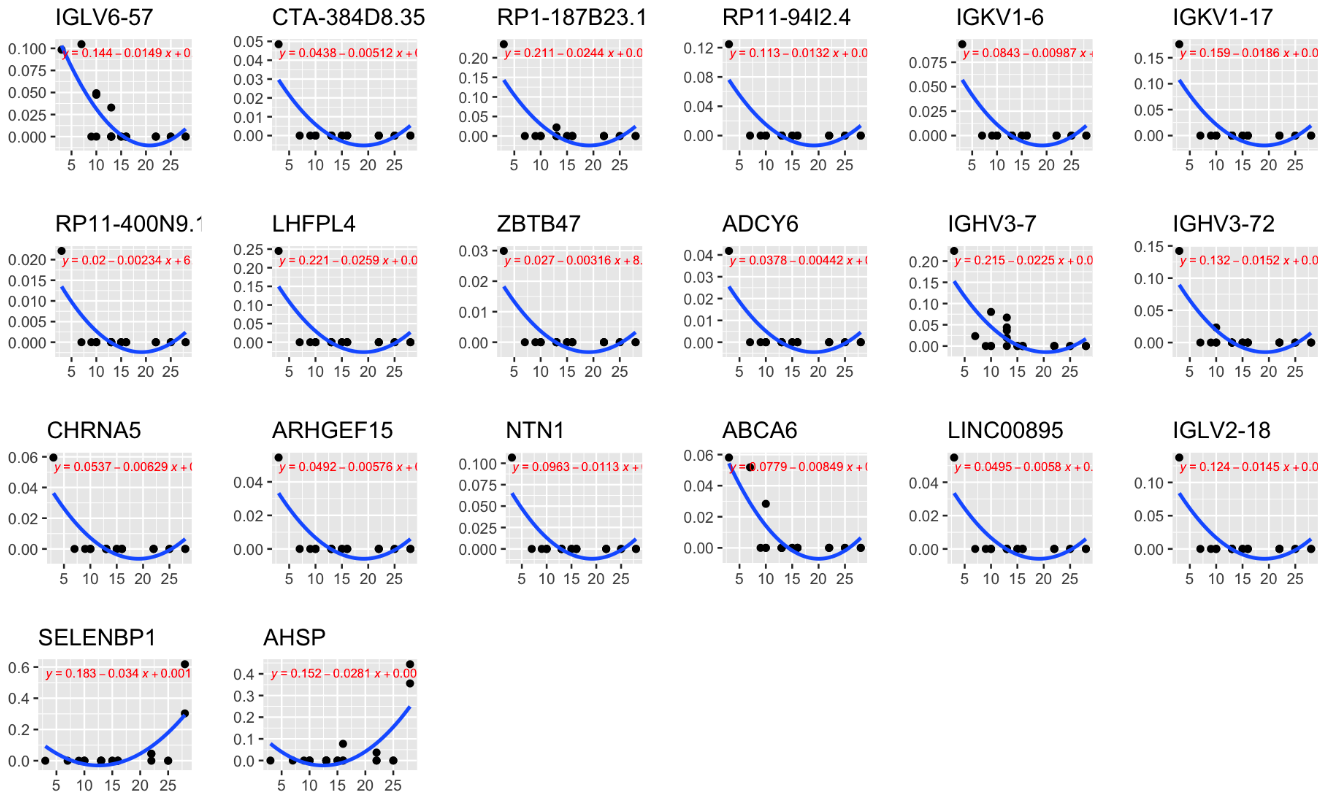
IGLV6-57
y = 0.144 – 0.0149 x + 0
CTA-384D8.35
y = 0.0438 – 0.00512 x +
RP1-187B23.1
y = 0.211 – 0.0244 x + 0.0
RP11-94I2.4
y = 0.113 – 0.0132 x + 0.0
IGKV1-6
y = 0.0843 – 0.00987 x
IGKV1-17
y = 0.159 – 0.0186 x + 0.0
RP11-400N9.
y = 0.02 – 0.00234 x + 6
LHFPL4
y = 0.221 – 0.0259 x + 0.0
ZBTB47
y = 0.027 – 0.00316 x + 8.
ADCY6
y = 0.0378 – 0.00442 x +
IGHV3-7
y = 0.215 – 0.0225 x + 0.0
IGHV3-72
y = 0.132 – 0.0152 x + 0.0
CHRNA5
y = 0.0537 – 0.00629 x +
ARHGEF15
y = 0.0492 – 0.00576 x +
NTN1
y = 0.0963 – 0.0113 x +
ABCA6
y = 0.0779 – 0.00849 x +
LINC00895
y = 0.0495 – 0.0058 x + 0.
IGLV2-18
y = 0.124 – 0.0145 x + 0.0
SELENBP1
y = 0.183 – 0.034 x + 0.001
AHSP
y = 0.152 – 0.0281 x + 0.00

d, Cycling T

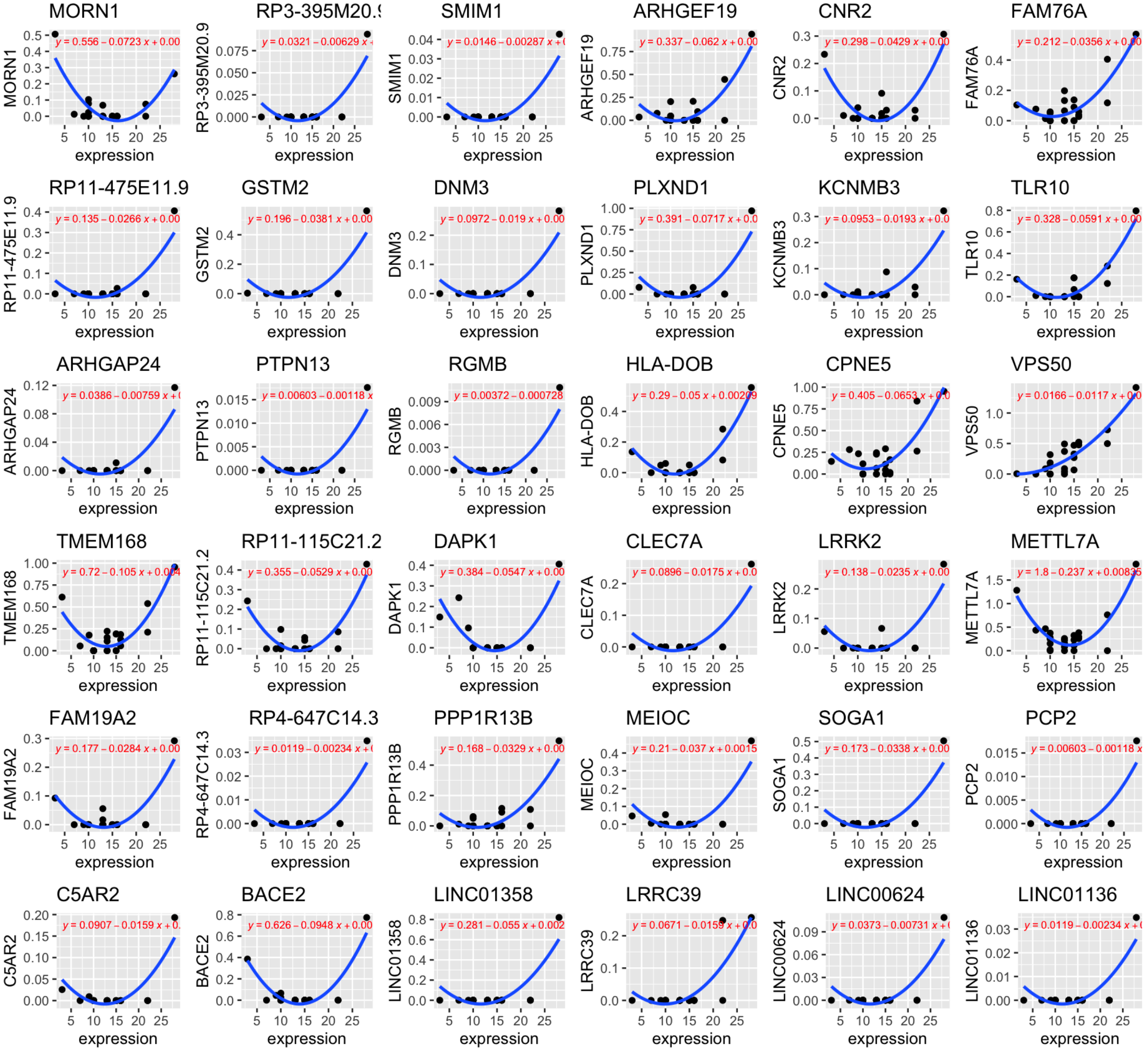
MORN1
RP3-395M20.9
SMIM1
ARHGEF19
CNR2
FAM76A
RP11-475E11.9
GSTM2
DNM3
PLXND1
KCNMB3
TLR10
ARHGAP24
PTPN13
RGMB
HLA-DOB
CPNE5
VPS50
TMEM168
RP11-115C21.2
DAPK1
CLEC7A
LRRK2
METTL7A
FAM19A2
RP4-647C14.3
PPP1R13B
MEIOC
SOGA1
PCP2
C5AR2
BACE2
LINC01358
LRRC39
LINC00624
LINC01136
expression

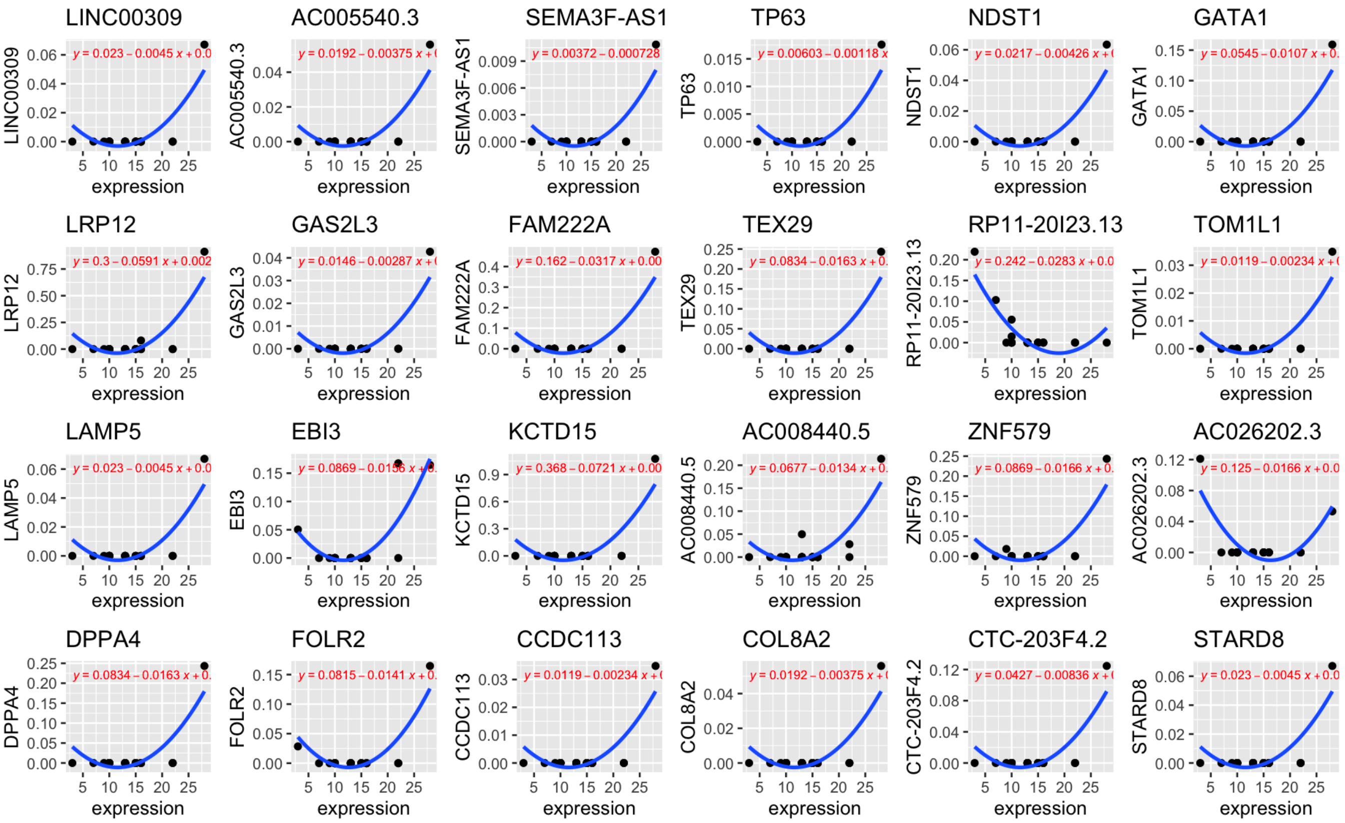
LINC00309
y = 0.023 − 0.0045 x + 0.0
AC005540.3
y = 0.0192 − 0.00375 x + (
SEMA3F-AS1
y = 0.00372 − 0.000728
TP63
y = 0.00603 − 0.00118 x
NDST1
y = 0.0217 − 0.00426 x + (
GATA1
y = 0.0545 − 0.0107 x + 0.
LRP12
y = 0.3 − 0.0591 x + 0.002
GAS2L3
y = 0.0146 − 0.00287 x + (
FAM222A
y = 0.162 − 0.0317 x + 0.00
TEX29
y = 0.0834 − 0.0163 x + 0.
RP11-20I23.13
y = 0.242 − 0.0283 x + 0.0
TOM1L1
y = 0.0119 − 0.00234 x + (
LAMP5
y = 0.023 − 0.0045 x + 0.0
EBI3
y = 0.0869 − 0.0166 x + 0.
KCTD15
y = 0.368 − 0.0721 x + 0.00
AC008440.5
y = 0.0677 − 0.0134 x + 0.
ZNF579
y = 0.0869 − 0.0166 x + 0.
AC026202.3
y = 0.125 − 0.0166 x + 0.0
DPPA4
y = 0.0834 − 0.0163 x + 0.
FOLR2
y = 0.0815 − 0.0141 x + 0.
CCDC113
y = 0.0119 − 0.00234 x + (
COL8A2
y = 0.0192 − 0.00375 x + (
CTC-203F4.2
y = 0.0427 − 0.00836 x + (
STARD8
y = 0.023 − 0.0045 x + 0.0
expression

e, Cytotoxic CD8 T

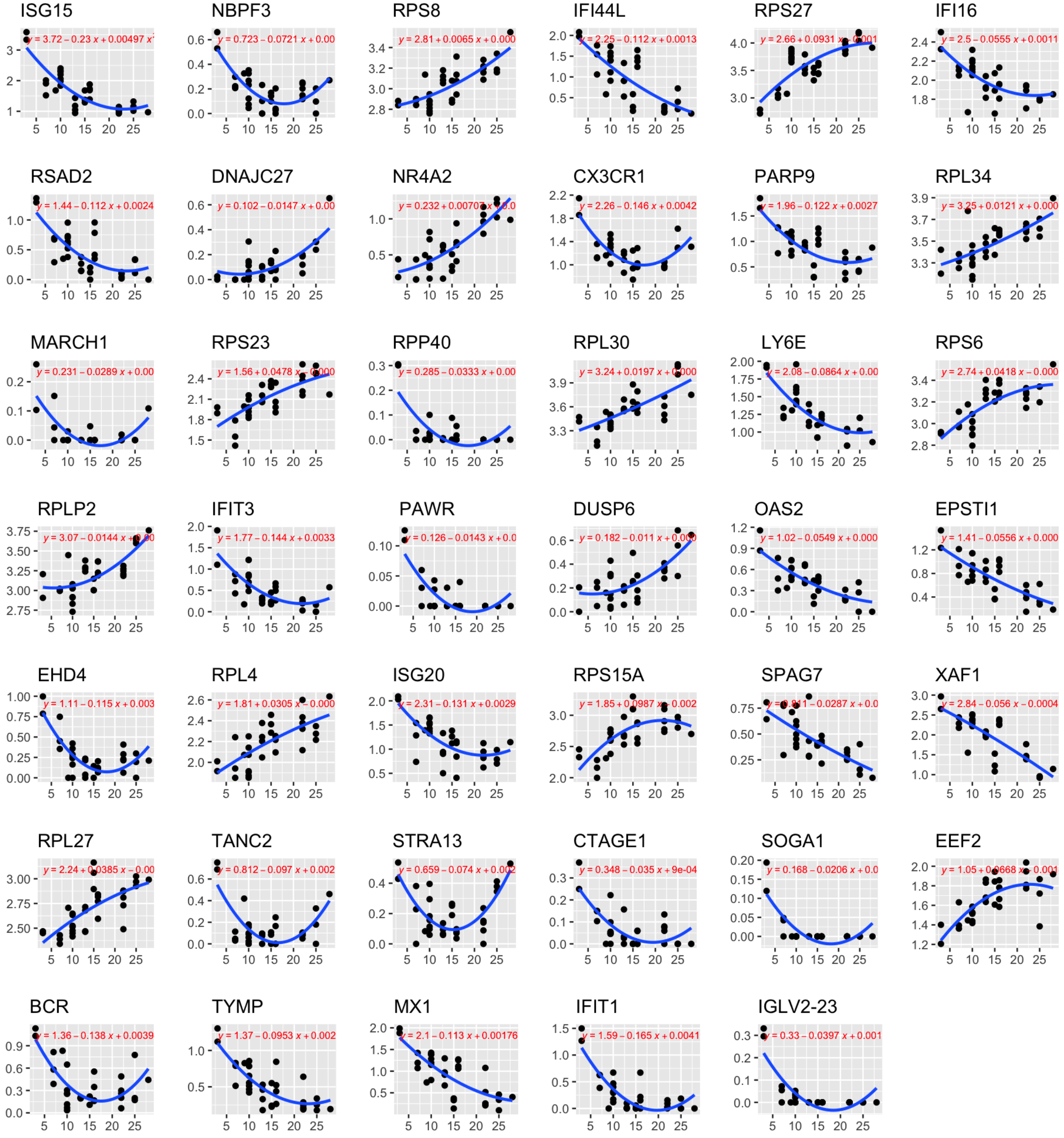

f, MAITs

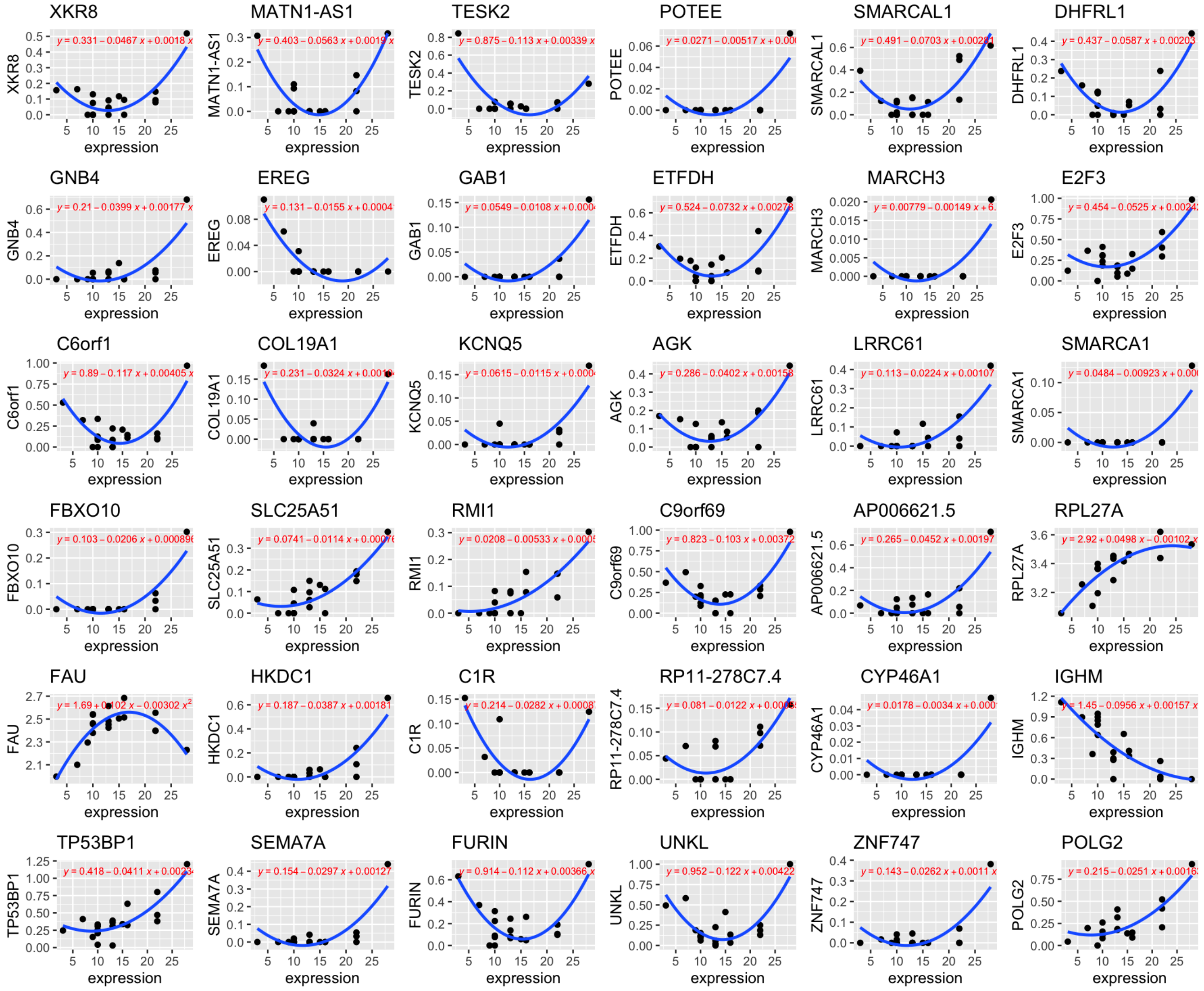
XKR8
MATN1-AS1
TESK2
POTEE
SMARCAL1
DHFRL1
GNB4
EREG
GAB1
ETFDH
MARCH3
E2F3
C6orf1
COL19A1
KCNQ5
AGK
LRRC61
SMARCA1
FBXO10
SLC25A51
RMI1
C9orf69
AP006621.5
RPL27A
FAU
HKDC1
C1R
RP11-278C7.4
CYP46A1
IGHM
TP53BP1
SEMA7A
FURIN
UNKL
ZNF747
POLG2
expression

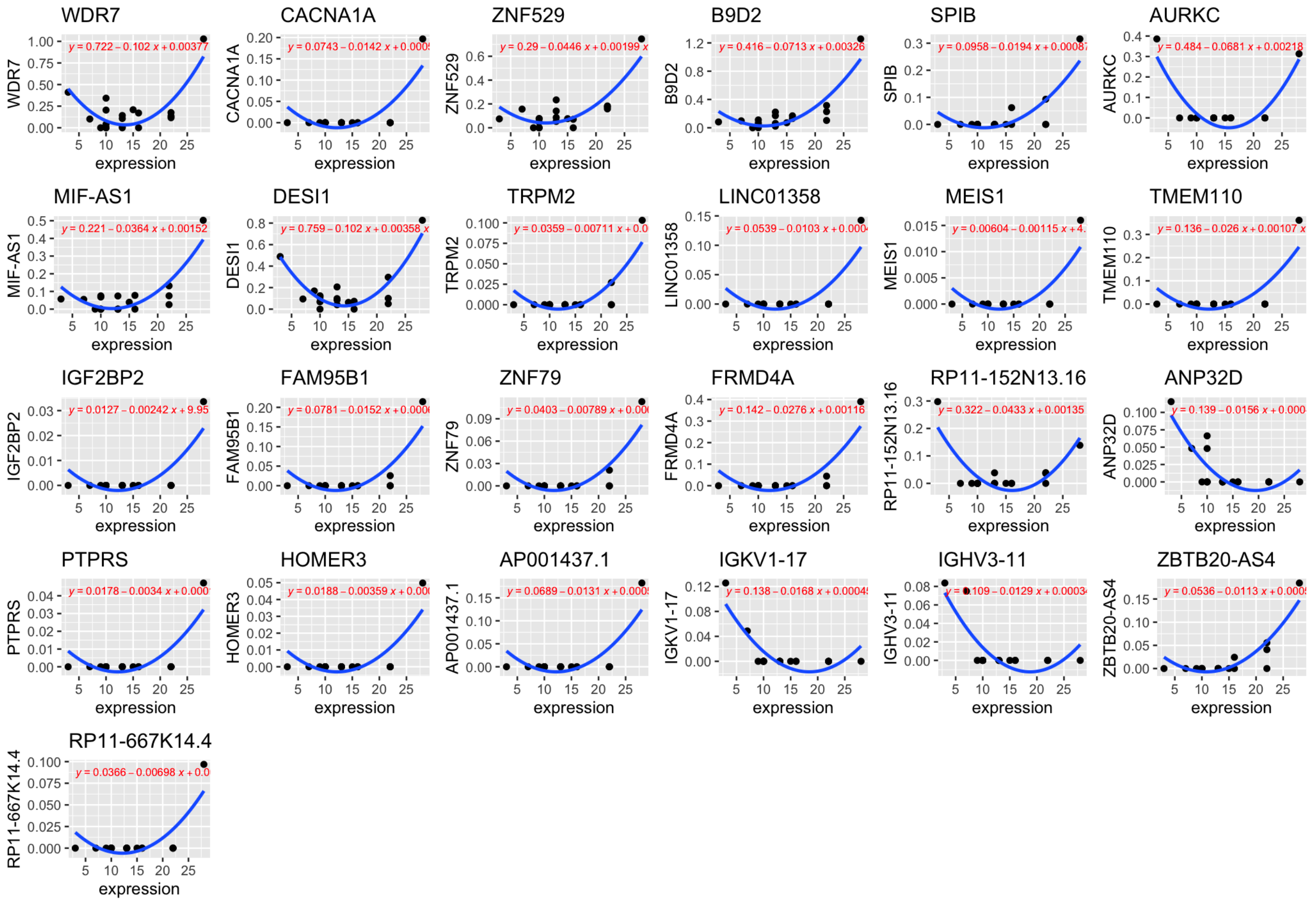

WDR7
y = 0.722 − 0.102 x + 0.00377
CACNA1A
y = 0.0743 − 0.0142 x + 0.000
ZNF529
y = 0.29 − 0.0446 x + 0.00199 x
B9D2
y = 0.416 − 0.0713 x + 0.00326
SPIB
y = 0.0958 − 0.0194 x + 0.0008
AURKC
y = 0.484 − 0.0681 x + 0.00218
MIF-AS1
y = 0.221 − 0.0364 x + 0.00152
DESI1
y = 0.759 − 0.102 x + 0.00358 x
TRPM2
y = 0.0359 − 0.00711 x + 0.0
LINC01358
y = 0.0539 − 0.0103 x + 0.000
MEIS1
y = 0.00604 − 0.00115 x + 4.
TMEM110
y = 0.136 − 0.026 x + 0.00107 x
IGF2BP2
y = 0.0127 − 0.00242 x + 9.95
FAM95B1
y = 0.0781 − 0.0152 x + 0.000
ZNF79
y = 0.0403 − 0.00789 x + 0.00
FRMD4A
y = 0.142 − 0.0276 x + 0.00116
RP11-152N13.16
y = 0.322 − 0.0433 x + 0.00135
ANP32D
y = 0.139 − 0.0156 x + 0.000
PTPRS
y = 0.0178 − 0.0034 x + 0.000
HOMER3
y = 0.0188 − 0.00359 x + 0.00
AP001437.1
y = 0.0689 − 0.0131 x + 0.000
IGKV1-17
y = 0.138 − 0.0168 x + 0.0004
IGHV3-11
y = 0.109 − 0.0129 x + 0.0003
ZBTB20-AS4
y = 0.0536 − 0.0113 x + 0.000
RP11-667K14.4
y = 0.0366 − 0.00698 x + 0.0
expression

g, memory B

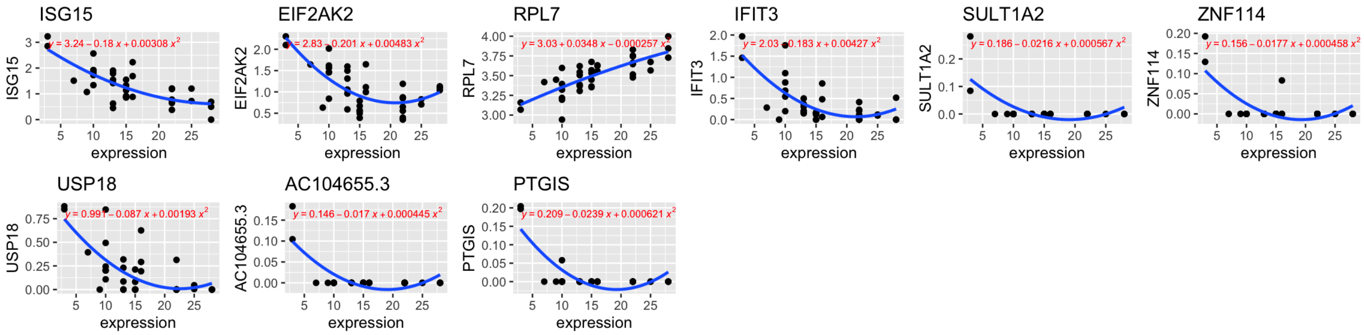

ISG15
y = 3.24 − 0.18 x + 0.00308 x²
EIF2AK2
y = 2.83 − 0.201 x + 0.00483 x²
RPL7
y = 3.03 + 0.0348 x − 0.000257 x²
IFIT3
y = 2.03 − 0.183 x + 0.00427 x²
SULT1A2
y = 0.186 − 0.0216 x + 0.000567 x²
ZNF114
y = 0.156 − 0.0177 x + 0.000458 x²
USP18
y = 0.991 − 0.087 x + 0.00193 x²
AC104655.3
y = 0.146 − 0.017 x + 0.000445 x²
PTGIS
y = 0.209 − 0.0239 x + 0.000621 x²
expression

h, NKs

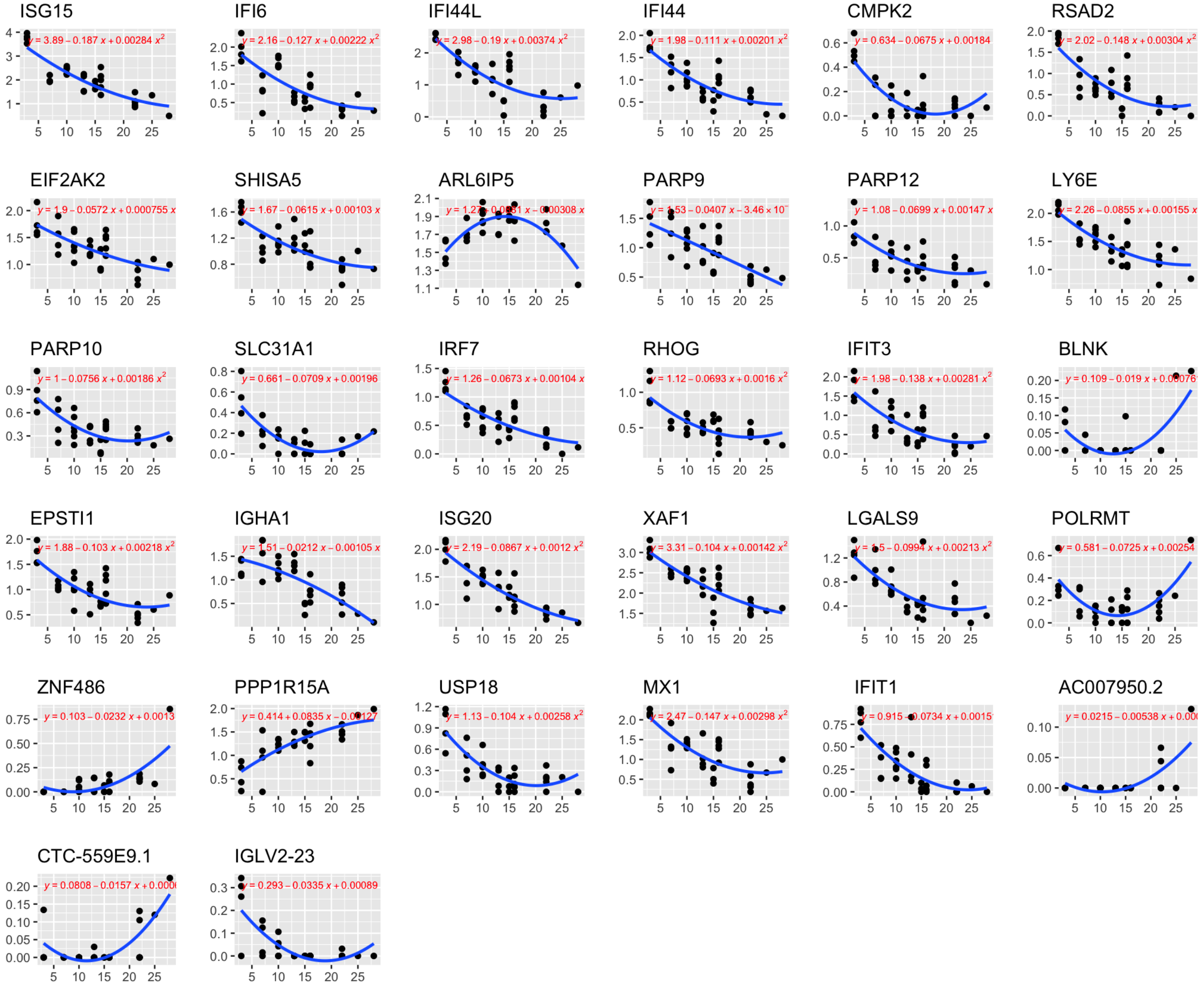

i, Plasma

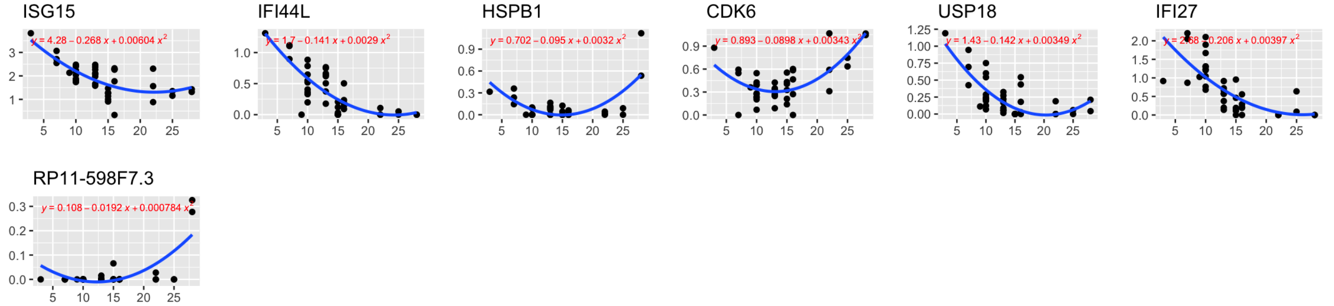

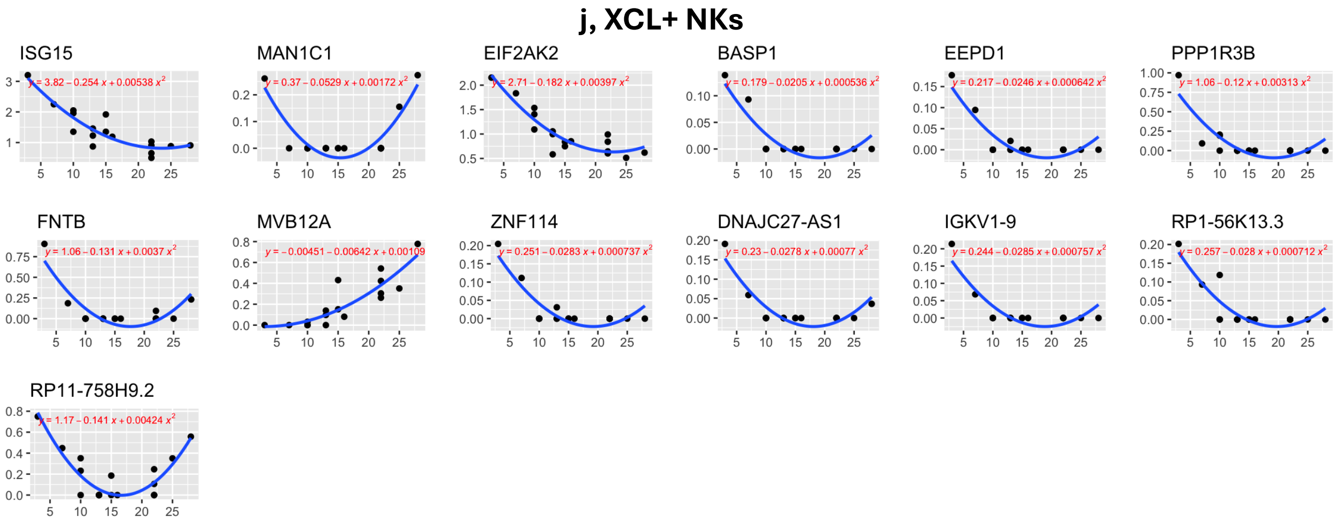

Supplementary Figure 3: sMetacell quadratic regression curves by cell type with formula for genes with FDR adjusted p < 0.05 and R^2 > 0.5. The plots are organized by cell type: **a)** Naïve T cells, **b)** Naïve B cells, **c)** Activated CD4 T cells, **d)** Cycling T cells, **e)** Cytotoxic CD8 T cells, **f)** MAITs, **g)** Memory B cells, **h)** NKs, **i)** Plasma, **j)** XCL+ NKs. Additional filtering of R^2>0.7 was applied to the cycling T cells to limit the number of overfit DEGs plotted. Otherwise, there would be too many plots.

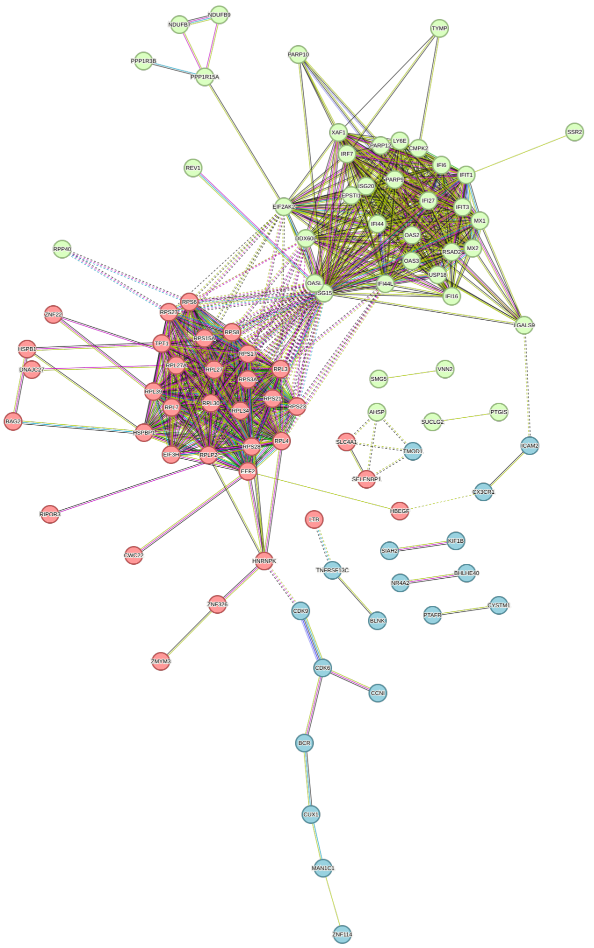

Supplementary Figure 4: STRING DB network colored by K-means clusters.

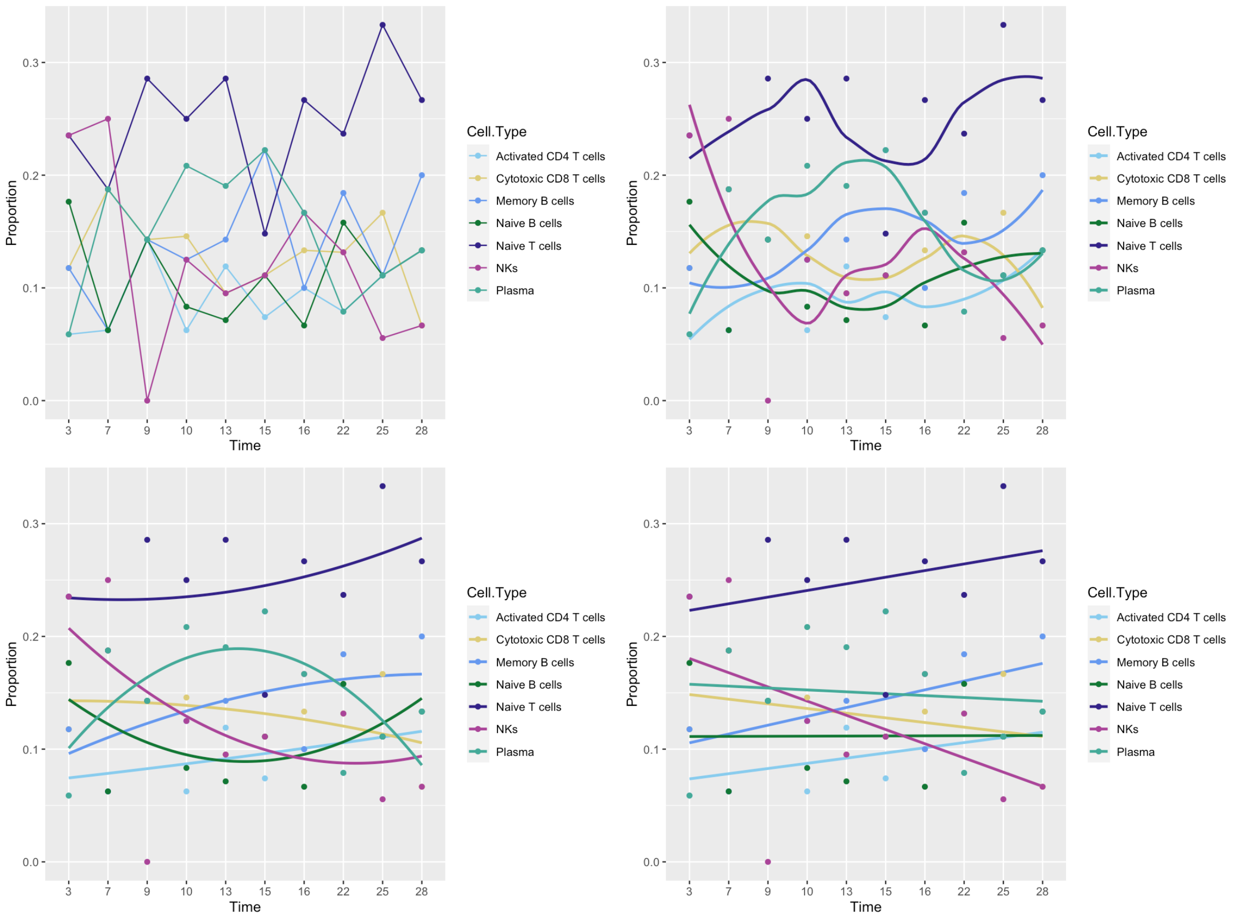

Supplementary Figure 5: sMetacell type proportions at individual time points, depicted with lines connecting points for no regression (top left), loess regression (top right), quadratic regression (bottom left), and linear regression (bottom right).
