# Supplementary material for: Metacell-based differential expression analysis identifies cell type specific temporal gene response programs in COVID-19 patient PBMCs
Source: NPJ Syst Biol Appl. 2024 Apr 5;10:36. doi: 10.1038/s41540-024-00364-2 (PMC10997786; doi:10.1038/s41540-024-00364-2)
Supplement: Supplementary file 3 — Reporting summary [file 41540_2024_364_MOESM3_ESM.pdf]

nature portfolio

Corresponding author(s): Deyou Zheng

Last updated by author(s): Feb 8, 2024

Reporting Summary

Nature Portfolio wishes to improve the reproducibility of the work that we publish. This form provides structure for consistency and transparency in reporting. For further information on Nature Portfolio policies, see our Editorial Policies and the Editorial Policy Checklist.

Statistics

For all statistical analyses, confirm that the following items are present in the figure legend, table legend, main text, or Methods section.

n/a	Confirmed	
☐	☒	The exact sample size (n) for each experimental group/condition, given as a discrete number and unit of measurement
☐	☒	A statement on whether measurements were taken from distinct samples or whether the same sample was measured repeatedly
☐	☒	The statistical test(s) used AND whether they are one- or two-sided<br>*Only common tests should be described solely by name; describe more complex techniques in the Methods section.*
☒	☐	A description of all covariates tested
☐	☒	A description of any assumptions or corrections, such as tests of normality and adjustment for multiple comparisons
☐	☒	A full description of the statistical parameters including central tendency (e.g. means) or other basic estimates (e.g. regression coefficient) AND variation (e.g. standard deviation) or associated estimates of uncertainty (e.g. confidence intervals)
☐	☒	For null hypothesis testing, the test statistic (e.g. F, t, r) with confidence intervals, effect sizes, degrees of freedom and P value noted<br>*Give P values as exact values whenever suitable.*
☒	☐	For Bayesian analysis, information on the choice of priors and Markov chain Monte Carlo settings
☒	☐	For hierarchical and complex designs, identification of the appropriate level for tests and full reporting of outcomes
☒	☐	Estimates of effect sizes (e.g. Cohen's d, Pearson's r), indicating how they were calculated

Our web collection on statistics for biologists contains articles on many of the points above.

Software and code

Policy information about availability of computer code

Data collection	Sample collection, sequencing, and quantification of gene counts were done by Zhu et al. 2020 (https://doi.org/10.1016/j.immuni.2020.07.009). No new experimental data was collected in the current study.
Data analysis	Custom code for data analysis is available on GitHub (https://github.com/bioinfoDZ/metacell_based_DEA). We used R version 4.3.2 and Python version 3.9. We used SEACells (v 0.3.3) and maSigPro (v 1.72).

For manuscripts utilizing custom algorithms or software that are central to the research but not yet described in published literature, software must be made available to editors and reviewers. We strongly encourage code deposition in a community repository (e.g. GitHub). See the Nature Portfolio guidelines for submitting code & software for further information.

Data

Policy information about availability of data

All manuscripts must include a data availability statement. This statement should provide the following information, where applicable:

- Accession codes, unique identifiers, or web links for publicly available datasets
- A description of any restrictions on data availability
- For clinical datasets or third party data, please ensure that the statement adheres to our policy

The data analyzed in the current study were described in a previous study (https://doi.org/10.1016/j.immuni.2020.07.009) and publicly available at the CNGB Nucleotide Sequence Archive (accession number: CNP0001102).

Research involving human participants, their data, or biological material

Policy information about studies with human participants or human data. See also policy information about sex, gender (identity/presentation), and sexual orientation and race, ethnicity and racism.

Reporting on sex and gender	We did not analyze sex or gender information in our manuscript. However, the original scRNA-seq data were from 2 male and 3 female patients, as described by Zhu et al. 2020 (https://doi.org/10.1016/j.immuni.2020.07.009).
Reporting on race, ethnicity, or other socially relevant groupings	We do not discuss race, ethnicity, or other socially relevant groupings in our manuscript.
Population characteristics	Patients used in this analysis were described in Zhu et al. 2020 (https://doi.org/10.1016/j.immuni.2020.07.009). Patients were all from Beijing, China. The ages of the five patients were 15, 45, 41, 44, and 85 years old.
Recruitment	We did not recruit any patients.
Ethics oversight	No ethics oversight required, as we didn't do the data collection.

Note that full information on the approval of the study protocol must also be provided in the manuscript.

Field-specific reporting

Please select the one below that is the best fit for your research. If you are not sure, read the appropriate sections before making your selection.

☒ Life sciences ☐ Behavioural & social sciences ☐ Ecological, evolutionary & environmental sciences

For a reference copy of the document with all sections, see nature.com/documents/nr-reporting-summary-flat.pdf

Life sciences study design

All studies must disclose on these points even when the disclosure is negative.

Sample size	Sample size for metacell analysis was based on the number of metacells for a particular time point or cell type, more than 3 in all cases.
Data exclusions	We excluded influenza patients (part of the original dataset we utilized) from our analysis since we were only interested in studying response to SARS-CoV-2 infection.
Replication	The analyzed data were from 5 patients.
Randomization	No applicable
Blinding	No applicablae

Reporting for specific materials, systems and methods

We require information from authors about some types of materials, experimental systems and methods used in many studies. Here, indicate whether each material, system or method listed is relevant to your study. If you are not sure if a list item applies to your research, read the appropriate section before selecting a response.

Materials & experimental systems

n/a	Involved in the study	
☒	☐	Antibodies
☒	☐	Eukaryotic cell lines
☒	☐	Palaeontology and archaeology
☒	☐	Animals and other organisms
☒	☐	Clinical data
☒	☐	Dual use research of concern
☒	☐	Plants

Methods

n/a	Involved in the study	
☒	☐	ChIP-seq
☒	☐	Flow cytometry
☒	☐	MRI-based neuroimaging

Plants

Seed stocks	N/A
Novel plant genotypes	N/A
Authentication	N/A
